# Genome sequencing and physiological characterization of three *Neoarthrinium moseri* strains

**DOI:** 10.1186/s12866-025-04274-z

**Published:** 2025-08-21

**Authors:** Nadine J. Hochenegger, Gabriel A. Vignolle, Matthias Schmal, Robert L. Mach, Astrid R. Mach-Aigner, Mohammad Javad Rahimi, Chin Mei Chan, Feng M. Cai, Irina S. Druzhinina, Christian Zimmermann

**Affiliations:** 1 Institute of Chemical, Environmental and Bioscience Engineering, TU Wien, Gumpendorfer Strasse 1a, Wien, 1060 Austria; 2 The Vatche and Tamar Manoukian Division of Digestive Diseases, Department of Medicine, David Geffen School of Medicine at UCLA, Los Angeles, CA USA; 3 Goodman-Luskine Microbiome Center, University of California, Los Angeles, CA USA; 4 Chemical Sciences, Faculty of Science, Universiti Brunei Darussalam, Bandar Seri Begawan, Jalan Tungku Link, Brunei Darussalam; 5 School of Ecology, Sun Yat-sen University, Shenzhen, 518107 China; 6 Royal Botanic Gardens, Kew Green, Richmond Surry TW9 3AE UK

**Keywords:** Amphisphaeriales, Ascomycota, Comparative genomics, Fungi, Genome mining, Microbiological assessment, Secondary metabolism

## Abstract

**Background:**

Fungi play essential ecological roles and have been utilized by humans for diverse applications such as industrial enzyme production or as sources of bioactive compounds. Recent research has highlighted the *Amphisphaeriales* order (*Ascomycota*) as promising producers of secondary metabolites of pharmaceutical importance. Within this family, the recently established genus *Neoarthrinium* includes species such as *N. brasiliense*,* N. lithocarpicola*,* N. moseri*,* N. trachycarpi*, and *N. urticae*. Existing literature has primarily focused on the taxonomy and phylogeny of *Neoarthrinium*, leaving its physiology, ecology, and metabolic potential unexplored.

**Results:**

This study presents the first investigation of the metabolic and genomic potential of *N. moseri*. We describe the isolation of two South-Asian *N. moseri* strains and the genome sequencing of these strains alongside the Colombian ex-type strain for the species. Comparative genome analysis reveals an exceptionally high number of biosynthetic gene clusters (BGCs), surpassing those of many other fungi in the *Amphisphaeriales* order. Additionally, the genome of *N. moseri* contains a diverse repertoire of carbohydrate-active enzymes (CAZymes), supporting its hypothesized ecological role as a phyllosphere fungus (putatively an endophyte and/or saprotroph). Ecophysiological assays, including BIOLOG phenotyping, demonstrate its ability to utilize a broad range of carbon sources, emphasizing ecological versatility.

**Conclusions:**

This study highlights *N. moseri* as a promising candidate for secondary metabolite discovery, providing foundational insights into the metabolic and genomic potential of the *Neoarthrinium* genus and related fungi.

**Supplementary Information:**

The online version contains supplementary material available at 10.1186/s12866-025-04274-z.

## Background

Fungi are a diverse kingdom with a broad range of ecological roles, e.g. decomposers of organic matter and symbiotic partners of plants – may it be in mutualistic and parasitic relations. Humankind has been using fungi for different purposes, such as food and feed fermentation, agricultural applications, enzymes production, and as source of bioactive compounds for medicine and industry [1]. The fungal secondary metabolism is generally considered to be a large untapped reservoir for novel bioactive compounds and drug leads [2, 3]. The ongoing efforts to find new pharmaceuticals encompass the search for new fungi and mining their genomes [4–6].

In the recent years, the order *Amphisphaeriales* (*Ascomycota*) gained increasing attention as promising secondary metabolite producers [7], especially, fungi related to such genera as *Apiospora* and *Arthrinium* (in the family of *Apiosporaceae*) [8, 9]. These fungi are known to produce a broad range of bioactive compounds, including cytochalasins with cytotoxic activity, polyketides such as apiosporic acid and hexylitaconic acid derivatives, and antimicrobial agents like apiosporamide. Additional compounds isolated from these genera exhibit antioxidant, tyrosinase-inhibitory, and quorum-sensing-disrupting properties. These findings highlight the chemical diversity of *Amphisphaeriales* and suggest that lesser-known genera such as *Neoarthrinium* may also harbor unexplored metabolic potential [7–9].

The genus *Neoarthrinium*, established in 2022 within the family *Apiosporaceae (vide infra)*, originally comprised four species: *N. lithocarpicola*, *N. moseri*, *N. trachycarpi*, and *N. urticae* [10]. These taxa were isolated from diverse terrestrial plant hosts across Asia and South America: *N. lithocarpicola* from diseased leaves of *Lithocarpus glaber* in China; *N. trachycarpi* from *Trachycarpus fortunei*, also in China; *N. moseri* from a dead plant in Colombia; and *N. urticae* from leaf litter in India. W. Gams isolated the *N. moseri* type strain CBS 164.80 from the dead petiole of *Mauritia minor* in Colombia in 1995 [11]. It was originally described as an unusual species of the genus *Wardomyces* (*Microascales*) until Jiang et al. realized that this isolate belongs to *Amphisphaeriales* and assigned it to *N. moseri* in 2022 [10]. The reclassification of *N. urticae* (syn. *Arthrinium urticae*) was based on sequence data from a single isolate, and its taxonomic placement remains tentative due to uncertainties regarding its representativeness [10]. Since 2022, three additional species have been described. *N. brasiliense*, added in 2024, further expanded the genus into South America [12]. In 2025, two ecologically and geographically distinct taxa were introduced: *N. lewisiae* was isolated from necrotic leaf spots on *Pandanus tectorius* (screwpine) in coastal Australia [13] and *N. aquaticum* was described from submerged plant tissue of the golden leather fern (*Acrostichum aureum*) in a freshwater habitat in Thailand [14], representing the first aquatic species in the genus. Notably, all *Neoarthrinium* strains have been isolated from the surface of plants [10–16]. Mukhopadhyay et al. also proposed the establishment of the new family *Neoarthriniaceae*, to accommodate the genus *Neoarthrinium* [14]. These findings highlight the ecological versatility of *Neoarthrinium*, encompassing both saprobic and potentially pathogenic lifestyles across tropical and subtropical regions, with substrates ranging from terrestrial leaf litter and petioles to aquatic ferns and coastal monocots.

Limited to mostly taxonomic and phylogenetic studies, existing literature provides little insight into the physiology, ecology, or metabolic potential of this genus. This study provides the first investigation of the metabolic and genomic potential of a species within the genus *Neoarthrinium*, namely *N. moseri*. We describe the isolation of two new *N. moseri* strains from Borneo and genome sequencing of these strains and the Colombian ex-type strain CBS 164.80. We have mined the genomes for carbohydrate-active enzymes (CAZymes) and biosynthetic gene clusters (BGCs) and compared them to other fungi in this regard. Further, we sequenced and annotated also the mitochondrial genome. We explored basic growth characteristics and substrate utilization of *N. moseri* to gain physiological insights using a BIOLOG Phenotype microarray. Additionally, we suggest that *N. trachycarpi* should not be considered a separate species but strains of *N. moseri* based on the genomic data together with already existing phylogenetics and a reassessment of the spore sizes.

## Methods

### Sampling and strain purification

The epiphytic fungi TUCIM 5799 and TUCIM 5827 were isolated from the same environmental sample, the adaxial surface of the healthy leaf of *Rubroshorea johorensis* (*Dipterocarpaceae*, *Malvales*; DNA BarCode maturase K (*matK*) deposited in NCBI GenBank MF993320.1 [17]), sampled in the high canopy (40–60 m above ground) of the lowland tropical rain forest surrounding the Kuala Belalong Field Studies Center (KBFSC, 4°32’48.2"N 115°09’27.9"E) located in the Temburong District of Brunei Darussalam (Borneo). For this purpose, the adaxial surface of a freshly sampled leaf was scratched by the sterile electric toothbrush (2 min) in 25 ml of sterile water supplemented with Tween-20 (0.01%) in large sterile Petri plate (20 cm in diameter). The resulting suspension was collected in 50 ml falcons and centrifuged at 4 °C for 15 min at 14 000 rpm. The resulting pellet was resuspended in 4 ml of sterile water and used for serial dilution and plating on potato dextrose agar (PDA, Carl Roth) supplemented with 200 mg/l of chloramphenicol. Young single spore fungal colonies were detected with the use of a stereo microscope and aseptically transferred to fresh PDA plates and cultivated at 28 °C in darkness, resulting in the isolation of TUCIM 5799 and TUCIM 5827. Agar plugs with pure mature cultures were preserved in 40% glycerol and stored at −80 °C in TU Wien Collection of Industrial Microorganisms (TUCIM).

### Maintenance and morphological characterization of strains

While the TUCIM strains were isolated in the course of this study, the CBS 164.80 strain was obtained from the CBS Filamentous fungi and Yeast Collection at the Westerdijk Fungal Biodiversity Institute. CBS 164.80, TUCIM 5827, and TUCIM 5799 were maintained on agar plates containing 30 g/l oatmeal (S-Budget, SPAR Österreichische Warenhandels-AG; shredded to ∅ 0.25 mm) and 15 g/l agar.

Three different media were used to initially evaluate the morphology of the three strains: MEA (20 g/l malt extract, 15 g/l agar), CYAS (Czapek yeast autolysate agar with 50 g/l NaCl; 3 g/l NaNO_3_, 5 g/l yeast extract, 30 g/l sucrose, 1.3 g/l K_2_HPO_4_,

0.5 g/l KCl, 0.5 g MgSO_4_ • 7 H_2_O, 0.01 g FeSO_4_ • 5 H_2_O, 0.005 g CuSO_4_ • 5 H_2_O,

15 g/l agar), and Oat (as above). 5 µl of spore solution.

(8 g/l NaCl, 0.05%(v/v) Tween-80) with an OD_600_ of 3.0 were applied to the middle of the agar plates, which were subsequently incubated at 28 °C for 13–14 days.

### Microscopy

Brightfield microscopy (BF) was performed using VWR Microscope TR 500 (VWR International GmbH, Darmstadt, Germany).

Scanning electron microscopy (SEM) of conidia was performed using COXEM EM-30AX PLUS with a SPT-20 Sputter. For sample preparation, conidia of the respective strain were softly scratched off an overgrown oatmeal-plate with a cotton swab. Conidia were then carefully distributed over a silver stripe which was attached to the stage of the device. Further proceedings were done according to the manufacturer’s instructions. Pictures were processed using the device’s own software Nanostation 3.0.4. The sizes of the spores were measured using FIJI (ImageJ2, Version 2.9.0).

### DNA extraction and library preparation

The *N. moseri* strains were cultivated in malt extract medium at 28 °C and 180 rpm for 10 days in an orbital shaking incubator. The biomass was filtered through miracloth (EMD Millipore Corp., Burlington, MA, USA), frozen in liquid nitrogen, lyophilized. For DNA extraction, the lyophilized biomass was disrupted using a Fast-Prep-24 (MP Biomedicals, Santa Ana/, CA, USA) with 0.37 g of glass beads ∅ 0.1 mm, 0.25 g of glass beads ∅ 1 mm, and a glass bead ∅ 5 mm at 6 m/s for 30 s. After the addition of 1 ml CTAB buffer (100 mM Tris.Cl, 20 mM EDTA, 1.4 M NaCl, 2% (w/v) CTAB, pH = 8.0) and 4 µl β-mercaptoethanol, the samples were subjected to two further disruption treatments on the Fast-Prep-24 at 5 m/s for 30 s and then incubated at 65 °C for 20 min. The supernatant was extracted with phenol, chloroform, isoamylalcohol (25:24:1) followed by a chloroform extraction. The supernatant was treated with RNase A (Thermo Fisher Scientific, Inc., Waltham, MA, USA) according to the manufacturer’s instructions. Finally, the DNA was precipitated with ethanol and dissolved in 10 mM Tris.Cl (pH = 8.0).

The DNA was sheared in a Diagenode Bioruptor^®^ Pico (Diagenode s.a., Liège, Belgium) with the settings set to high and three cycles of 15 s “on” and 60 s “off”. The sheared DNA was purified using PCR purification kit (Thermo Fisher Scientific, Inc., Waltham, MA, USA) and then double side size selected with “NEBNext Ultra™ sample purification beads” (New England Biolabs, Ipswich, MA, USA) for 800 bp fragments. The library preparation was performed following the protocol of “NEBNext^®^ Ultra™ II DNA Library Kit with Purification Beads” and “NEBNext^®^ Multiplex Oligos for Illumina (Index Primer Set1 and Set2)” (New England Biolabs, Ipswich, MA, USA). The average size in bp of the library was measured with the fragment analyzer from Advanced Analytical Technologies using the Agilent dsDNA 915 Reagent Kit (35–5000 bp) and analyzed with the PRO size software (Agilent Technologies, Santa Clara, California, USA). The exact DNA concentrations were measured with an “invitrogen™ Qubit™ fluorometer” in ng/µl (Thermo Fisher Scientific, Inc., Waltham, MA, USA) using a “Quant-iT™ dsDNA BR Assay” kit (Thermo Fisher Scientific, Inc., Waltham, MA, USA). Specifically, two libraries were created with a DNA fragment length of 1293 ± 6 bp and 1136 ± 7 bp, the average DNA concentrations were 34.93 ± 0.25 ng/µl and 10.63 ± 0.23 ng/µl, resulting in a 40.899 nM and a 14.178 nM library, respectively. The libraries were diluted to the appropriate 4 nM concentration for sequencing.

### Sequencing

The sequencing of the *N. moseri* library was performed on an Illumina MiSeq platform using two V3 Reagent Kit (600 cycles) and one V2 Nano Reagent Kit (500 cycles) following the standard protocol of Illumina sequencing protocol without adding PhiX control to the runs (Illumina, San Diego, California, USA), resulting in a total of 67,670,936 paired end-reads. The raw data were deposited at the Sequence Read Archive (SRA) under the accession SRR13570309 (CBS 164.80), SRR13747339 (TUCIM 5827) and SRR13747338 (TUCIM 5799). The quality profiles and all further figures, if not specified otherwise, were visualized in R [18].

### Extracting the mitochondrial genome and cleaning the raw reads

First, a preliminary assembly was performed using SPAdes v3.13.1 [19] with default parameters for each strain separately. Mitochondrial sequences were identified in each strain by performing a sequence similarity analysis using BLAST [20] (non-redundant nucleotide database). Contigs ranging from 500 to 1000 bp were then used as seed input for NOVOplasty v3.7 [21] to extract the whole circularized mitochondrial genome of *N. moseri* CBS 164.80, TUCIM 5799 and TUCIM 5827. This was performed in an iterative manner. The mitochondrial genomes were visualized with CGViewer [22]. The mitochondrial genomes were annotated with the automated MITOS2 web pipeline. The mitochondrial genomes were deposited at GenBank with accession no. MW554918 (CBS 164.80), MW660808 (TUCIM 5827), and MW660809 (TUCIM 5799).

Using the mitochondrial genomes of the strains as input an index was built with bowtie v1.2.2 [23], respectively, and the mitochondrial flagged reads were extracted using --un option from each raw reads file. The clean raw reads were then re-paired with Fastq-pair [24] to use paired end read assemblers.

### Whole - genome assembly

For each strain respectively, the raw cleaned paired end reads were quality trimmed using Trimmomatic [25] in the command line and specifying PE for paired end reads and ILLUMINACLIP: Adapter-PE.fa:2:30:10:2:keepBothReads LEADING:3 TRAILING:3 SLIDINGWINDOW:4:15 MINLEN:36 to ensure high quality adapter-free reads. Then the cleaned raw reads were assembled using SPAdes v3.13.1 [19], for each strain separately. Furthermore, the high quality trimmed cleaned paired end reads were used for scaffolding with SSPACE-Standard v3.0 in an iterative manner with following command line options -x 1 -m 50 -o 20 -k 8 -a 0.70 -n 30 -z 150 –b and –k 6. Ns introduced during the assemblies and the scaffolding, so called gaps, were closed with GapFiller v1-10 [26] using following commands -m 30 -o 6 -r 0.7 -n 10 -d 50 -t 10 -g 0 -i 5 -b.

The assemblies were further improved by using Pilon v1.21 [27] iteratively. We first indexed the assemblies with bwa [28], SAMtools v1.7 [29] and picard [30]. The high quality trimmed cleaned paired end reads were mapped to the matching indexed assemblies of the individual *N. moseri* strains with bwa. The reads were mapped and combined in one step. Next, we sorted and created bam files from the sam files using SAMtools. Together with the paired sequencing reads, these were used as input for Pilon to iteratively improve each genome.

The genome assemblies were deposited at GenBank the accession no. GCA_022829205.1 (CBS 164.80), GCA_022829195.1 (TUCIM 5799), and GCA_022829225.1 (TUCIM 5827).

### Phylogenetic analysis

To confirm the taxonomic identity of the two newly isolated strains (TUCIM 5799 and TUCIM 5827), we performed molecular identification using a multi-locus sequence analysis based on the internal transcribed spacer (ITS), the large subunit ribosomal RNA gene (LSU), and the beta-tubulin gene (*tub2*). Sequences used for the multiple sequence alignment can be seen in Table 1. Multiple sequence alignment was performed by using MAFFT [31]. The alignments for each gene were manually curated and concatenated using MEGA [32]. Based on this MSA iqtree2 was used to generate phylogenetic trees using the “K2P + I” nt substitute model [33]. Trees were generated by applying 1000 bootstraps in 10 individual runs each. Numbers at nodes indicate bootstrap support values in %. The tree was visualized using figtree software [34].

**Table 1:** Isolates and GenBank accession numbers used in the phylogenetic analyses. NA, not available

		GenBank accession number		
Species	Strain	ITS	LSU	tub2
*Neoarthrinium moseri*	CBS 164.80	LN850995	LN851049	LN851154
*Neoarthrinium moseri*	TUCIM 5799	Additional File 1		
*Neoarthrinium moseri*	TUCIM 5827	Additional File 1		
*Neoarthrinium urticae*	IMI 326,344	AB220245	AB220339	NA
*Neoarthrinium trachycarpi*	CFCC 53,038	MK301098	NA	MK303394
*Neoarthrinium trachycarpi*	CFCC 53,039	MK301099	NA	MK303395
*Neoarthrinium lithocarpicola*	CFCC 54,456	ON427580	ON427582	ON456914
*Neoarthrinium lithocarpicola*	CFCC 55,883	ON427581	ON427583	ON456915
*Neoarthrinium brasiliense*	URM 8364	OQ540770	OQ540773	OQ473592
*Neoarthrinium brasiliense*	V150.1	OQ540771	OQ540774	OQ473593
*Neoarthrinium aquaticum*	P4A41	PQ481181	PQ481182	PQ639434
*Neoarthrinium lewisiae*	BRIP72527g	PV364379	PV364384	NA
*Lepteutypa fuckelii* (outgroup)	CBS 140,409	NR_154123	KT949902	MH554677

### Gene prediction

To predict the genes, we first masked the repetitive elements in the nuclear genomes of *N. moseri* CBS 164.80 and our two new isolates to reduce the number of false positives during the subsequent gene prediction using RepeatMasker [35] Further, we performed an tRNA prediction with tRNAscan-SE v1.3.1 [36] using the unmasked genome. tRNAscan-SE.

For the gene prediction, we used Augustus v3.3.2 [44], because no transcriptome data was available. Augustus v3.3.2 [37] was trained with the genome of *Pestalotiopsis fici* (assembly PFICI; BioSample accession: SAMN02369365) following the protocol by Hoff & Stanke [38]. The genomes and the gene sets were evaluated using Quast v5.0.2 [39]. Quast v5.0.2 includes benchmarking with Benchmarking Universal Single-Copy Orthologs (BUSCO) v3.0.2, this was performed with the eukaryote dataset of 303 BUSCOs from 100 species. We further evaluated the gene predictions by aligning the amino acid sequences using Blastp v2.9.0+ [20] against the UniProt database [40].

### Annotation

The gene sets were first annotated using Blastp against the UniProt protein database. Protein ANNotation with Z-scoRE (PANNZER2) [41] was used to provide both GO and free text DE producing an accurate functional annotation. CAZymes were annotated using the dbCAN2 [42] meta server by applying a HMMer (Hidden Markov model) search [43], a DIAMOND [44] search and a Hotpep [45] search and combining the three outputs.

### BGC Genome mining and comparison

The antiSMASH 7.1.0 fungal-version web version [46] was used for genome mining for secondary metabolite BGCs with following extra features applied: KnownClusterBlast, ClusterBlast, SubClusterBlast, MIBiG cluster comparison, ActiveSiteFinder, RREFinder, Cluster Pfam analysis, Pfam-based GO term annotation, TIGRFam analysis (Table 2).

**Table 2:** Genomes used for comparative antismash genome mining

Organism	Strain	GenBank
*Aspergillus flavus*	NRRL3357	GCA_014117465.1
*Aspergillus fumigatus*	Af293	GCA_000002655.1
*Fusarium oxysporum*	Fo47	GCA_013085055.1
*Apiospora malaysiana*	STlab-iicb	GCA_006508115.1
*Hypoxylon fragiforme*	CBS 206.31	GCA_022984875.1
*Microdochium bolleyi*	J235TASD1	GCA_001566295.1
*Pestalotiopsis fici*	W106-1-2	GCA_000516985.1
*Poronia punctata*	CBS 180.79	GCA_022579005.1
*Rosellinia necatrix*	CMW50482	GCA_026420105.1
*Xylaria longipes*	CBS 148.73	GCA_025201785.1

The detected BGCs within the *N. moseri* genomes were further compared to each other using BiG-SLiCE v2.0.0 [47, 48] (with the HMM database ‘bigslice-models-2022-11-30’) under default feature extraction, clustering settings, cutoffs, and GCF clustering thresholds. Additionally, BiG-SCAPE v1.1.8 [49] was used for comparative analysis, employing the latest release of the Pfam-A.hmm database [50] (processed with hmmpress from HMMER), with a distance threshold of 0.3 for GCF clustering and MIBiG comparison enabled.

### Ecophysiological profiling

MEX-medium (30 g/l malt extract, 1 g/l peptone, 15 g/l agar) was used as basis to investigate growth of the three *N. moseri* strains under different stress conditions.

The strains were incubated at different temperatures (37 °C, 28 °C, and 21 °C) in order to narrow down the possible optimal growth temperature of the strains.

For the purpose of testing the tolerance to increasing salinity, NaCl was added to the MEX plates to a final concentration of 0, 0.5, 1, 1.5, 2, and 2.5 M, respectively.

Additionally, the tolerance of the strains to varying pH in the medium was tested. Therefore, the MEX plates were adjusted to pH 2, 3, 5, 7, 8, and 9 with HCl and NaOH, respectively, under sterile conditions. 10 g/l Phytagel + 5 mM MgCl_2_ was used instead of agar in those plates. The plates used for salinity- and pH-tolerance-testing were incubated at 28 °C. All plates were inoculated by applying 5 µl of a spore solution (OD_600_ of 3) to the center of the plates and were incubated at the according temperatures for 11 days.

The resulting colony radii were measured using FIJI (ImageJ2, Version 2.9.0).

### BIOLOG assay

Growth of *N. moseri* strains on 95 different carbon sources was performed using the BIOLOG FF (Filamentous Fungi) MicroPlate™ (Art.Nr. 1006) panels (Biolog, Hayward, CA, United States). Spore solution was applied to FF Inoculating Fluid (Art.Nr. 72106) to a turbidity-increase of 20% (Turbidity Deice Name) and incubated for 16 days at 28 °C. Fungal growth was determined by measuring optical density at 750 nm (OD_750_) using a plate reader (TECAN Spark^®^ Multimode Microplate Reader) after each 24 h, starting after inoculation (day 0). The assay was performed in technical triplicates. We compared OD_750_max independent from growth rate as a simple method to estimate the potential biomass formation on carbon sources. Data was evaluated using GraphPad Prism 9.1.2 (GraphPad Software, LLC.). Statistics were performed using GraphPad Prism 9.1.2 (GraphPad Software, LLC.).

## Results

### *Isolation and DNA Barcoding of two epiphytic* N. moseri *strains*

In 2008, we isolated the two epiphytic fungi TUCIM 5799 and TUCIM 5827 from the adaxial surface of the healthy high canopy leaf of *Shorea johorensis* (*Dipterocarpaceae*, *Malvales*; DNA BarCode maturase K (*matK*) deposited in NCBI GenBank MF993320.1 [17]) on Borneo.

**Fig. 1:**
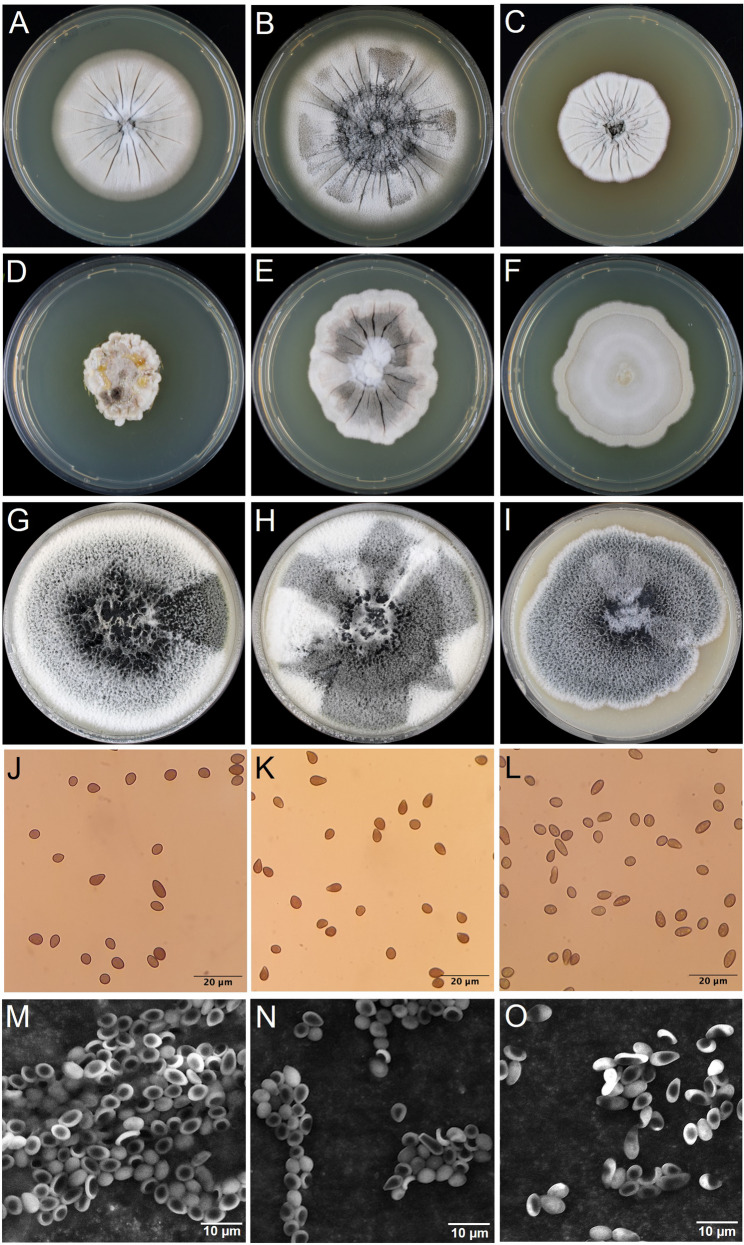
Morphology of *N. moseri* CBS 164.80 (A, D, G), TUCIM 5799 (B, E, H), and TUCIM 5827 (C, F, I) on malt extract (A-C), Czapek yeast autolysate (D-F), and oatmeal (G-I) plates after incubation at 28 °C for 14 days. Brightfield and scanning electron microscopy of spores of *N. moseri* CBS. 164.80 (J, M), TUCIM 5799 (K, N), and TUCIM 5827 (L, O).

The two new isolates as well as the reference strain CBS 164.80 form a light-colored mycelium (beige on malt extract and Czapek yeast autolysate plates, white on oatmeal after 14 days, Fig. 1A-I). The texture of the mycelia and the size of the colonies differ amongst the strains and depend on the culture media. Further, we observed the formation of a large quantity of conidia on oatmeal plates. The conidia were present in a slimy layer on the surface of the colonies. The strain TUCIM 5799 also produced smaller amounts of conidia on malt extract and Czapek yeast autolysate plates. The conidia of the two new isolates and the *N. moseri* reference strain look alike: They are melanized, dark colored, and are most frequently pear-shaped, with a length of 4.2–6.7 μm long and a width of 3–3.9 μm, in contrast to essentially larger dimensions previously reported for this species [11] (Fig. 1J-O; Table 3) and more consistent with the values obtained for *N. trachycarpi* (6.1–8.5 × 4.2–5.3 μm) [15]. Thus, we re-assessed Gams’ SEM images [11], and measured actually similar sizes we obtained from our own picture (Table 3).

**Table 3:** Average conidia dimensions

Strain	Length [µm]	Width [µm]	Reference
*N. moseri* CBS 164.80	10–14	3–4.5	[11] (original values)
*N. moseri* CBS 164.80	4.5–7.1	3.1–4.3	Re-assessment of pictures from [11]
*N. moseri* CBS 164.80	4.4–6	3.1–4.1	This publication
*N. moseri* TUCIM 5799	4.2–5.7	3.2–3.8	This publication
*N. moseri* TUCIM 5827	4.3–6.7	3–3.9	This publication
*N. trachycarpi*	6.1–8.5	4.2–5.8	[15]
*N. lithocarpicola*	5–8.5	4.5–6	[10]
*N. brasiliense*	4–5	3–4	[12]
*N. aquaticum*	8–13	6–10	[14]
*N. urticae*	n/a	n/a	
*N. lewisiae*	n/a	n/a	

**Fig. 2:**
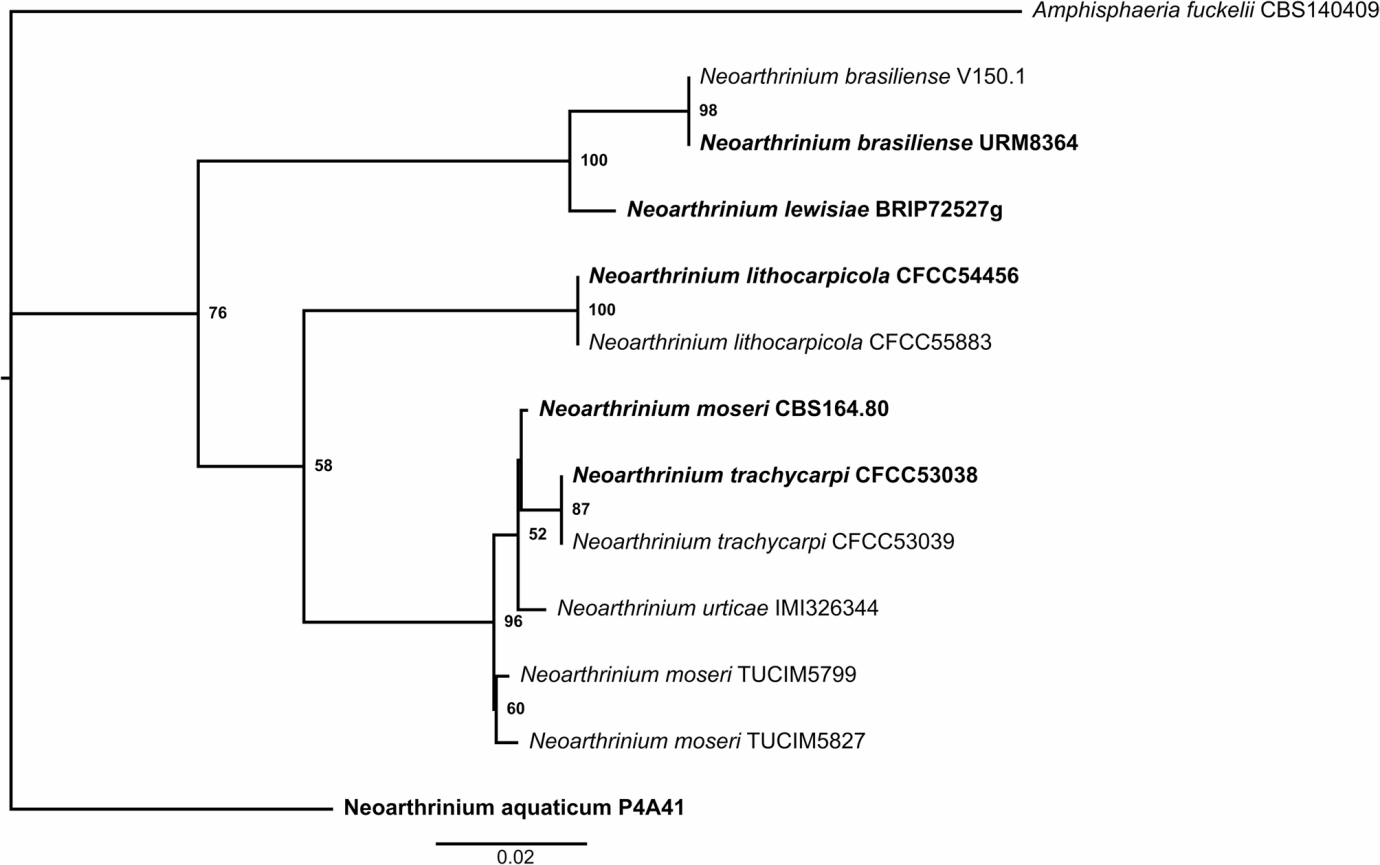
Phylogenetic tree based on the concatenated multiple sequence alignment of ITS, LSU and *tub2 *of the indicated fungal isolates (type strains in bold)*.* The rooted phylogenetic tree is the consensus of 10 individual runs applying 1000 bootstraps utilizing the maximum-likelihood approach. Values at nodes indicate bootstrap support values in %.

To confirm the classification of the new isolates as *N. moseri* strains, we performed a multiple sequence alignment of the available ITS, LSU and *tub2* sequences of the *Neoarthrinium* strains and constructed a phylogenetic tree (Fig. 2). *N. moseri* CBS 164.80 clusters together with two *N. trachycarpi* strains, *N. urticae* and the two new isolates, with the two Borneo isolates forming a distinct subclade (Fig. 2). Notably, all pairwise comparisons within the *N. moseri* clade showed very high sequence identity across the ITS, LSU, and *tub2* loci, generally exceeding 99%, with the lowest observed identity being 97.77% (Additional File 2).

### Mitochondrial and nuclear genome of N. moseri

To assess the genetic makeup and potential metabolic capabilities of *N. moseri*, we sequenced and assembled the complete mitochondrial and nuclear genomes of three strains using Illumina paired-end sequencing followed by SPAdes assembly, with additional refinement steps including NOVOPlasty for mitochondria and Pilon for genome polishing. The extracted circularized mitochondrial genomes have a length of 42,769 bp, 43,978 bp, and 42,769 bp and with GC contents of 27.52%, 27.52%, and 27.53% for the strain CBS 164.80, TUCIM 5799, and TUCIM 5827, respectively (Fig. S1). The respective average sequencing coverages were at 364x, 8,939x, and 464x.

The size of *N. moseri* nuclear genomes is between 43.7 Mbp and 46.1 Mbp with average sequencing coverages between 32x and 141x. The detailed results of the genomes and assembly characteristics (size, GC content, characteristics for scaffold number and size, N_50_ and L_50_) are summarized in Table S1. To evaluate the completeness of the genome assembly, we performed a Benchmarking Universal Single-Copy Orthologues (BUSCO) analysis with the eukaryote dataset [51]. 100% complete BUSCOs without duplicates were found in all three assemblies (Table S1). Further, we calculated the average nucleotide identity (ANI) and found the three strains to be highly similar (Table S2). Additionally, the genomes of the three strains exhibited a similar GC content of around 52.7%, and masked element analysis indicated a low level of repetitive sequences, with simple repeats and low complexity regions occupying less than 1% of the genomes.

### Gene prediction and annotation

First, we identified and masked the repetitive elements in the nuclear genomes of the three *N. moseri* strains (Table 4). Additionally, we performed a tRNA prediction and found a total of 196, 190 and 189 tRNA genes, respectively (Table 4, Additional Files 3–5). The analysis of repetitive elements revealed that less than 1% of each *N. moseri* genome was composed of transposable elements or low-complexity regions, indicating a relatively compact genome architecture with few repetitive sequences. Among the identified elements, short interspersed nuclear elements (SINEs) and long interspersed nuclear elements (LINEs) are known to be non-coding mobile elements that can influence genome evolution and gene regulation. Simple repeats and low complexity regions often play roles in genome structure, microsatellite formation, or replication slippage but are typically underrepresented in compact fungal genomes. The consistent tRNA gene counts (189–196) across strains suggest a conserved translational capacity among the three sequenced *N. moseri* strains.

**Table 4:** Masked repetitive elements and tRNA genes found in the genomes of the *N. moseri* strains CBS 164.80 (CBS), TUCIM 5799 (5799) and TUCIM 5827 (5827)

Masked element	Number of elements*			Length occupied in bp			Percentage of sequence		
Strain	CBS	5799	5827	CBS	5799	5827	CBS	5799	5827
SINEs	35	35	33	2,289	2,404	2,231	0.01%	0.01%	-
LINEs	223	222	220	16,838	17,399	16,757	0.04%	0.04%	0.04%
LTR elements	4	3	3	300	204	200	-	-	-
DNA elements	50	49	55	3,751	3,598	4,260	0.01%	0.01%	0.01%
Unclassified	1	1	1	142	142	72	-	-	-
Small RNA	86	78	74	12,181	12,157	11,815	0.05%	0.03%	0.05%
Simple repeats	7,572	7,432	7,532	306,538	294,925	297,695	0.70%	0.66%	0.64%
Low complexity	652	600	652	30,991	27,670	30,995	0.06%	0.06%	0.07%
tRNA	196	189	190	17,154	16,788	16,884	0.04%	0.04%	0.04%

As no transcriptome data were available, the gene prediction was performed on the masked genome using a model trained with the genome of *P. fici*. We obtained approx. 14,000 genes for *N. moseri* (Table 5). A significant portion of the predicted genes (34.4–36.7%) did not match any sequences in public databases below the E^−5^ threshold (Table 5), suggesting the presence of potentially novel genes unique to *N. moseri*.

**Table 5:** Gene predictions

Strain	Predicted putative genes	genes without BLAST hits below E^−5^
CBS 164.80	13,929	4,797 (34.4%)
TUCIM 5799	14,160	4,964 (35.0%)
TUCIM 5827	14,595	5,352 (36.7%)

The predicted gene sets were annotated by blasting them against the UniProt database and via the PANNZER2 web interface. The combined functional annotations are given in Additional Files 6–8.

### Genome mining for CAZymes

**Fig. 3:**
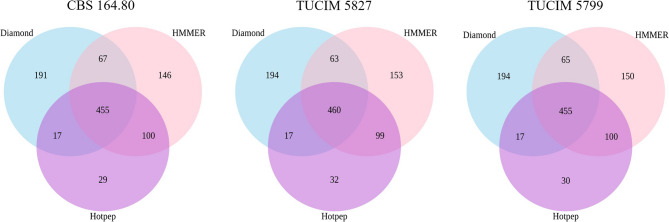
The genomes of the three sequences *N. moseri* strains were mined for putative CaZymes using Diamond, HMMER, and Hotpep.

The ability to decompose organic matter and the saprotrophic lifestyle are hallmarks of fungal biology. Fungi thrive on plant biomass and other natural materials by degrading complex and simple carbohydrates using so-called carbohydrate active enzymes (CAZymes) [52]. We used dbCAN2 (a meta-server for CAZyme annotation) and a HMMer (Hidden Markov model) search [43], a DIAMOND search [44], and a Hotpep search [45] to predict the CAZymes in the three *N. moseri* genomes (Fig. 3; Table 6). In total, 1,005, 1,011, and 1,018 CAZymes were predicted by all three methods in CBS 164.80, TUCIM 5799, and TUCIM 5827, respectively (Fig. 3, Additional Files 9–11, including 455, 455, and 460 genes predicted by all three methods (Fig. 3).

The dbCAN2 server also predicts certain subclasses of CAZyme. Glycosyltransferases (GT families) catalyze glycosidic bond formation and inversion and are part of the posttranslational modification steps in different compound formation processes. Glycoside hydrolases (GH families) is a large group of enzyme families which hydrolyse glycosidic bonds. Carbohydrate esterases (e.g., CE1, CE10 families) catalyze de-N or de-O-acylation of ester bonds in saccharides like in pectin. Polysaccharide lyases (e.g., PL1, PL7 families) cleave polysaccharide chains via β-elimination. Redox enzymes with auxiliary activities are involved in the breakdown processes of polysaccharides and lignin. The respective numbers of the predicted CAZymes subclasses (*sensu* dbCAN2) are also listed in Table 6. For comparative purposes, we selected *P. fici* as a reference because it belongs to the same order (*Amphisphaeriales*) and has a publicly available, well-annotated genome with CAZyme predictions. While broader comparative analyses across more taxa could offer additional insight, our focus here was to provide a contextually relevant benchmark from a phylogenetically close relative.

**Table 6:** The carbohydrate active enzymes (CAZymes) found with dbCAN2 a meta-server for cazyme annotation. Glycosyltransferases (GT); glycoside hydrolases (GH); carbohydrate esterases (CE); polysaccharide lyases (PL); redox enzymes with auxiliary activities (AA)

Strain	Total	GT	GH	CE	PL	AA
*N. moseri* CBS 164.80	1005	148	476	93	27	222
*N. moseri* TUCIM 5799	1011	152	479	94	27	222
*N. moseri* TUCIM 5827	1018	151	476	95	27	231
*P. fici*	-	121	460	138	39	-

### Genome mining for secondary metabolites

**Fig. 4:**
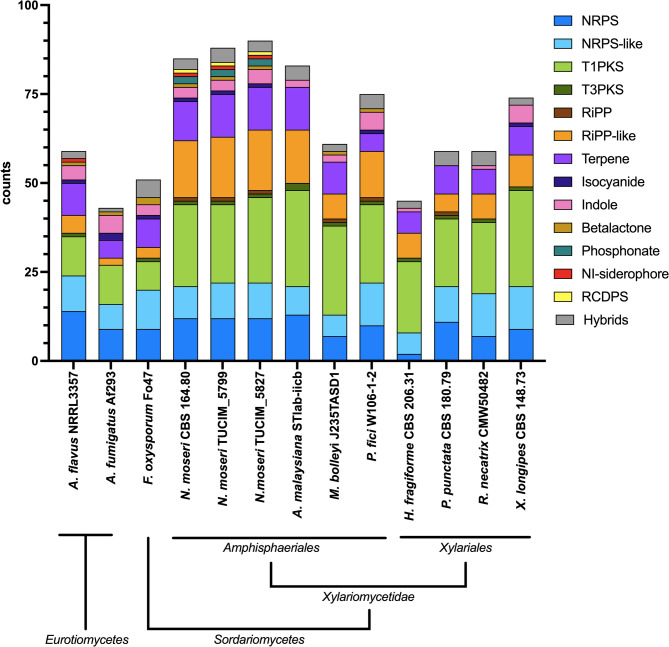
Overview of the predicted BGCs (antiSMASH 7.0) in the genomes of the indicated fungal species, including the three sequenced *N. moseri *strains (*Amphisphaeriales* genus* incertae sedis*). NRPS, non-ribosomal peptide synthetase; PKS, polyketide synthase; RiPP, ribosomally synthesized and post-translationally modified peptides; RCDP, arginine-containing cyclodipeptide synthase; hybrids, BGCs that contain core enzymes with characteristics for different classes, e.g. PKS-NRPS fusion enyzmes.

We used antiSMASH [46] to mine the genomes of the three *N. moseri* strains for genes that might be involved in the production of secondary metabolites and compared them to a few fungi of the same order (*Amphisphaeriales*) and the sister-taxon, the *Xylariales*, as well as the proficient secondary metabolite-producers *Aspergillus flavus*, *A. fumigatus* (*Eurotiales*) [53] and *Fusarium oxysporum* species complex (*Hypocreales*) [54]. Notably, the three *N. moseri* strains exhibited the highest BGC-count among the compared strains (85 in CBS 164.80, 88 in TUCIM 5799, 90 in TUCIM 5827) (Fig. 4, Additional File 12).

Next, we compared the identified BGCs in three *N. moseri* strains using BiG-SLiCE (Additional Files 13 and 14). A total of 244 BGCs were used as input. The analysis grouped them into 88 Gene Cluster Families (GCFs). The majority of GCFs (76) contained matching BGCs from all three strains, reflecting high conservation of biosynthetic loci within the species. Eight GCFs were exclusively found in one strain, and four GCFs were shared between two strains. Notably, the singleton GCFs were predominantly RiPP-like BGCs (Additional File 14), suggesting limited conservation or lower comparability in this class.

To validate and complement these results, we additionally performed a BiG-SCAPE analysis on the same set of BGCs (Additional file 15). The results were largely consistent with the BiG-SLiCE output. One previously ungrouped RiPP BGC could be matched to a homologous pair, while another singleton BGC was found to be part of a larger, split BGC locus that had been predicted as two separate BGCs by antiSMASH (Additional File 14). Interestingly, among all detected BGCs, only one GCF could be associated with a known MIBiG cluster, namely the scytalone/T3HN BGC (Additional File 14).To get a better understanding of the secondary metabolite potential, we manually compared the predicted BGCs of the *N. moseri* strains to the MIBIG 4.0 database [55] and assessed the predictions by a manual BGC comparison using the cblaster tool [56] if the BGC contained more than one gene (Additional File 16). We found 14 BGCs similar to previously characterized BGCs (Additional File 17). Additional to BGCs for common compounds, such as siderophores or choline, we also found BGCs for antibacterial and antifungal substances, such as citridone A and related compounds, fusaric acid, and (-)-mellein. We also found BGCs highly similar to BGCs reported from plant pathogenic fungi, e.g. brassicicene C and koraiol, and the plant growth hormone gibberellin. Further, we detected BGCs for pharmaceutically interesting compounds such as the histone deacetylase inhibitor depudecin, the immunomodulator swainsonine, and the cytotoxin eupenifeldin.

### Growth optima and stress tolerance

**Fig. 5:**
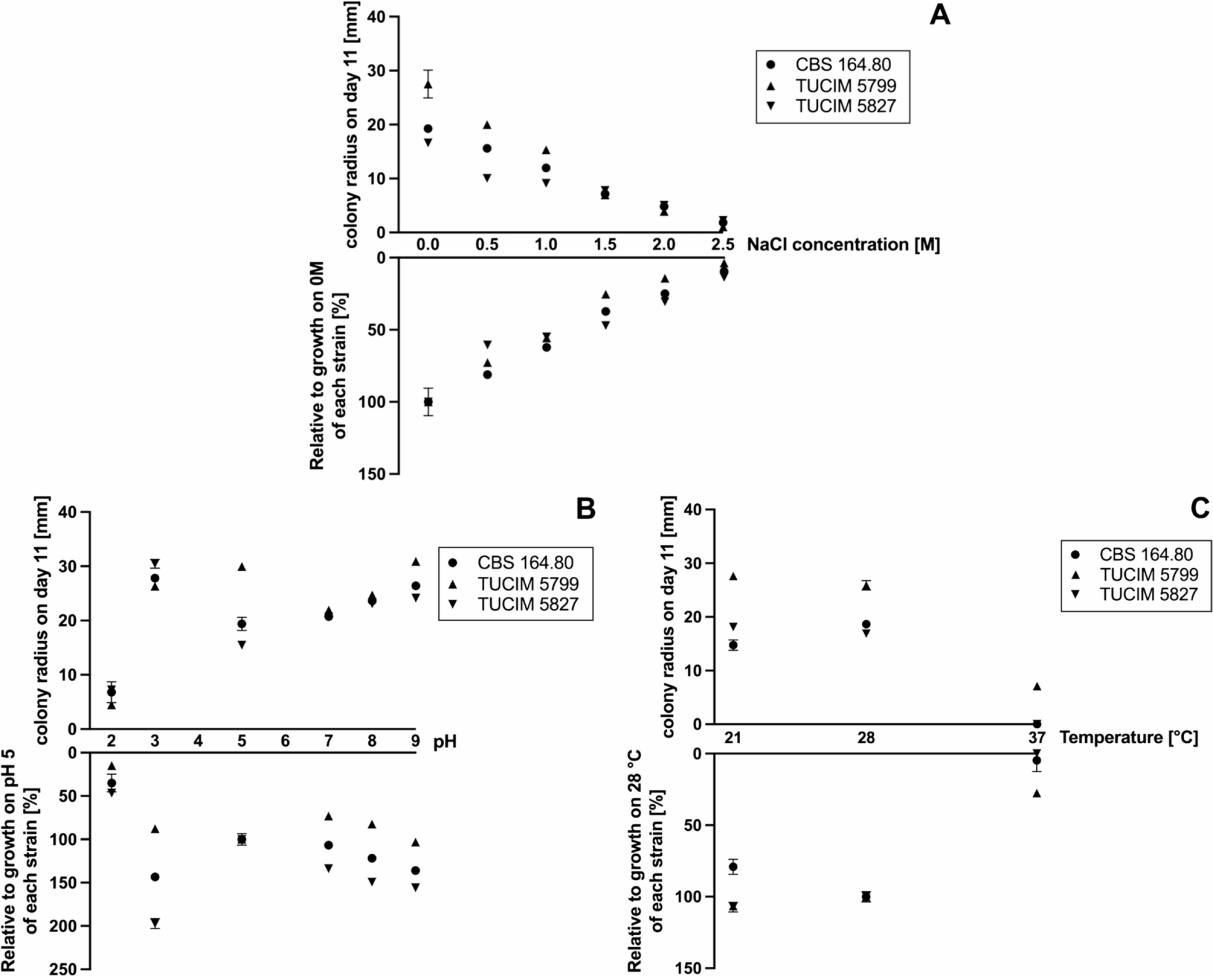
Growth response of the*N. moseri* strains to different NaCl-concentrations (A), pH (B), and temperatures (C), respectively, after 11 days of incubation on MEX. Data shows the mean of three experiments ± SD. Corresponding statistics are provided in Additional File 18.

To gain some insights into the ecophysiology of *N. moseri*, we cultivated the three strains on malt extract plates (MEX) with varying NaCl concentrations, pH values, or at different temperatures (Fig. 5A-C, individual growth curves are depicted in Fig. S2). All three *N. moseri* strains grew on MEX with NaCl concentrations ranging from 0 to 2.5 M (Fig. 5A), with optimal growth at 0 M and the least at 2.5 M. Mycelial growth progressively decreased as NaCl concentration increased.

All three *N. moseri* strains grew on the media adjusted to any of the pH values between 2 and 9 (Fig. 5B), with pH 2 as the least tolerated condition. All three strains grew comparatively well at pH 3 with CBS 164.80 and TUCIM 5827 having their optima at this condition. In contrast, TUCIM 5799 has its growth optimum at pH 5, which was the second-least favorite condition for the other two strains. Regarding pH 7, 8, and 9, the three strains behaved similar to each other and showed modest growth. The optimum in the alkaline pH range was surprisingly at pH 9.

All three *N. moseri* strains grew at 21 °C and 28 °C (Fig. 5C). There was no difference in growth of TUCIM 5799 and TUCIM 5827 between these incubation temperatures. CBS 164.80 grew slightly better at 28 °C than at 21 °C. Only TUCIM 5799 was able to tolerate 37 °C but showed poor growth.

Although all three *N. moseri* strains showed robust growth under a wide range of conditions, notable physiological differences were observed. For example, TUCIM 5799 was the only strain to grow, albeit weakly, at 37 °C, indicating a slightly higher thermotolerance compared to the other strains (Fig. 5C). Under acidic conditions, CBS 164.80 and TUCIM 5827 exhibited optimal growth at pH 3, whereas TUCIM 5799 grew best at pH 5. These observations suggest some degree of strain-specific adaptation to environmental pH.

### Utilization of different carbon sources

**Fig. 6:**
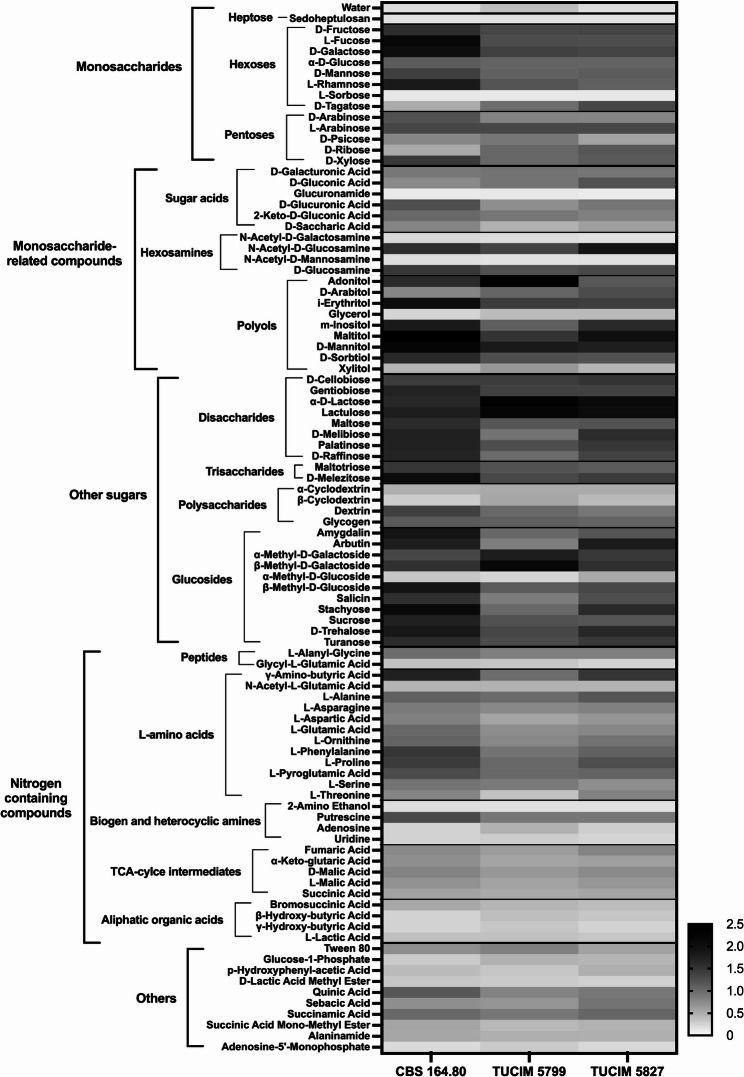
Comparative heatmap of the OD_750_max of the three tested *N. moseri* strains on the indicated carbon sources in BIOLOG FF microplates. Grayscale indicates respective OD_750_max.

We performed a BIOLOG Phenotype microarray to assess the carbon source utilization profiles of the three *N. moseri* strains. The growth was monitored over 16 days, and we used the maximal produced biomass throughout the growth period (OD_750_max) for comparison (Fig. 6; Table 7). The individual growth curves on each carbon source are shown in the supplements (Fig. S3-S10). The carbon sources were grouped as described previously [57]. “No growth” indicates OD_750_max values less than or equal to the respective OD_750_max on water. Neither of the three *N. moseri* strains grew on sedoheptulosan, L-sorbose, glucuronamide, N-acetyl-D-mannosamine, and 2-amino ethanol. TUCIM 5827 did not grow on N-acetyl-galactosamine and α-methyl-D-glucoside, either. On all other tested carbon sources we observed differently strong growth, suggesting that *N. moseri* has an adaptive and diverse primary metabolism.

All three *N. moseri* strains showed strong growth on saccharides (Fig. 6). In contrast, nitrogen-containing compounds were poorly utilized. For CBS 164.80, we observed strong growth only on γ-amino-butyric acid, L-phenylalanine, and L-proline. TUCIM 5827 grew well only on γ-amino-butyric acid and TUCIM 5799 did not metabolize any of the tested nitrogen-containing compounds well (Table 7).

The carbon source utilization assay further revealed subtle but meaningful differences among the strains. Notably, CBS 164.80 and TUCIM 5827 grew strongly on γ-aminobutyric acid (GABA), whereas TUCIM 5799 showed only moderate growth (Table 7). As GABA is a stress-related signaling compound in plants, this utilization pattern may indicate adaptive metabolic flexibility in response to host- or habitat-derived compounds. Additionally, TUCIM 5827 exhibited consistently lower biomass production across many carbon sources, suggesting reduced growth vigor or a more specialized metabolic strategy.

**Table 7:** Growth of the *N. moseri* strains on different carbon sources. Strong growth was defined as an OD_750_max of 15% above the median OD_750_max for each strain, weak growth as 15% below the median OD_750_max, medium in between these two thresholds

Carbon-Source	CBS 164.80	TUCIM_5799	TUCIM_5827	Carbon-Source	TUCIM_5799	TUCIM_5827	
Water	no			Monosaccharide-related compounds: Sugar acids			
Monosaccharides: Heptose				D-Galacturonic Acid	medium		
Sedoheptulosan	no			D-Gluconic Acid	medium		
Monosaccharides: Hexoses				Glucuronamide	no		
D-Fructose	strong	medium	strong	D-Glucuronic Acid	medium		
L-Fucose	strong		medium	2-Keto-D-Gluconic Acid	medium		
D-Galactose	strong			D-Saccharic Acid	slight		
α-D-Glucose	medium			Monosaccharide-related compounds: Hexosamines			
D-Mannose	medium			N-Acetyl-D-Galactosamine	slight	no	
L-Rhamnose	strong		medium	N-Acetyl-D-Glucosamine	strong		
L-Sorbose	no			N-Acetyl-D-Mannosamine	no		
D-Tagatose	slight	medium	strong	D-Glucosamine	strong		
Monosaccarides: Pentoses				Monosaccharide-related compounds: Polyols			
D-Arabinose	medium			Adonitol	strong	medium	
L-Arabinose	medium	strong		D-Arabitol	medium	strong	
D-Psicose	medium		slight	i-Erythritol	strong		
D-Ribose	slight	medium		Glycerol	slight		
D-Xylose	strong	medium		m-Inositol	medium	strong	
				Maltitol	strong		
				D-Mannitol	strong		
				D-Sorbitol	strong	medium	
				Xylitol	medium	slight	
Other sugars: Disaccharides				Other sugars: Glucosides			
D-Cellobiose	strong			Amygdalin	strong	medium	
Gentiobiose	strong			Arbutin	strong	medium	strong
α-D-Lactose	strong			α-Methyl-D-Galactoside	medium	strong	
Lactulose	strong			β-Methyl-D-Galactoside	strong		
Maltose	strong		medium	α-Methyl-D-Glucoside	slight		no
D-Melibiose	strong	medium	strong	β-Methyl-D-Glucoside	strong		
Palatinose	strong			Salicin	strong	medium	strong
D-Raffinose	strong	medium	strong	Stachyose	strong	medium	strong
Other sugars: Trisaccharides				Sucrose	strong		medium
Maltotriose	strong		medium	D-Trehalose	strong		
D-Melezitose	strong			Turanose	strong		
Other Sugars: Polysaccharides				Nitrogen containing compounds: Peptides			
α-Cyclodextrin	slight			L-Alanyl-Glycine	medium		
β-Cyclodextrin	slight	medium	slight	Glycyl-L-Glutamic Acid	slight		
Dextrin	medium						
Glycogen	medium						
Nitrogen containing compounds: L-amino acids				Nitrogen containing compounds: TCA-Cylce Intermediates			
γ-Amino-butyric Acid	strong	medium	strong	Fumaric Acid	medium		
N-Acetyl-L-Glutamic Acid	slight			α-Keto-Glutaric Acid	medium		slight
L-Alanine	medium			D-Malic Acid	medium		
L-Asparagine	medium			L-Malic Acid	medium		
L-Aspartic Acid	medium			Succinic Acid	slight		
L-Glutamic Acid	medium			Nitrogen containing compounds: Aliphatic Organic Acids			
L-Ornithine	medium			Bromosuccinic Acid	slight		
L-Phenylalanine	strong	medium		β-Hydroxy-butyric Acid	slight		
L-Proline	strong	medium		γ-Hydroxy-butyric Acid	slight		
L-Pyroglutamic Acid	medium				slight		
L-Serine	medium			Others			
L-Threonine	medium	slight	medium	Tween 80	medium		slight
Nitrogen containing compounds: Biogene and Heterocyclic Amines				Glucose-1-Phosphate	slight		
2-Amino Ethanol	no			p-Hydroxyphenyl-acetic Acid	slight		
Putrescine	medium			D-Lactic Acid Methyl Ester	slight		
Adenosine	slight			Quinic Acid	medium		
Uridine	slight			Sebacic Acid	medium		
				Succinamic Acid	medium		
				Succinic Acid Mono-Methyl Ester	slight		
				Alaninamide	slight		
				Adenosine-5’-Monophosphate	slight		

## Discussion

In this study, we isolated and characterized two new strains of *N. moseri* (TUCIM 5799 and TUCIM 5827). Morphological and genetic analyses confirmed the attribution of these strains to *N. moseri*, while also revealing strain-specific variations in growth and morphological characteristics. *N. moseri* exhibited high adaptability across a range of growth conditions, including varying pH levels and salt concentrations, and demonstrated a diverse and adaptable carbon utilization pattern. Genome mining further uncovered an exceptional number of BGCs, highlighting the species’ considerable secondary metabolism potential. Additionally, an in-depth analysis of CAZymes revealed a rich repertoire consistent with a saprotrophic lifestyle, suggesting *N. moseri* plays a significant role in decomposing plant materials in its natural environment.

### Phylogeny and Taxonomic Insights

The type strain of *N. moseri* (CBS 164.80) was isolated in Colombia in 1995 and originally described as an unusual *Wardomyces* [11]. In 2022, Jiang et al. created the genus *Neoarthrinium* upon isolation of several new fungi in China [10].

Importantly, *N. moseri*, *N. trachycarpi*, and *N. urticae* possess highly similar ITS, LSU, and *tub2* sequences (. 2), as already discussed by Jiang et al. and Mukhopadhyay et al. [10, 14]. However, Jiang et al. proposed keeping *N. moseri* and *N. trachycarpi* as separate species based on conidial size differences. Importantly, they compared their own measurements of *N. trachycarpi* to the values of *N. moseri* as reported by W. Gams. Our reassessment of Gams’ SEM images [11], along with our own conidial size measurements of the CBS strain and our two new isolates, revealed inconsistencies with previously reported values. It appears the spores of *N. moseri* and *N. trachycarpi* are similar in size (Table 3), suggesting that *N. trachycarpi* is not a distinct species. Further, the high ANI values among the CBS strain and our isolates (Table S2) strongly suggest that they belong to the same species. This fact, taken together with the phylogenetic tree based on the ITS, LSU, and *tub2* sequences (Fig. 2) and the analysis by Mukhopadhyay et al. [14]. suggest that *N. moseri*, *N. trachycarpi*, and *N. urticae* belong to the same species.

Notably, the high ANI values among the sequenced *N. moseri* strains also indicate a stable genomic architecture and strong relatedness across the strains, despite spatiotemporal differences in their isolation.

The available sequences for *N. urticae* are from a single isolate from leaf litter in India (IMI 326344) but not from isolates from the type host *Urtica dioica* L. (*Urticaceae*). Jiang et al. already stated that “Additional molecular studies on verified isolates from *Urtica* collected in Europe are necessary to reveal whether IMI 326344 represents true *N. urticae*. However, *N. urticae* appears to be very rare and we are unaware of any additional collections with the exception of the type.” [10]. We second this opinion. Resolving these taxonomic uncertainties will also require more comprehensive sampling, sequence datasets, including less-conserved regions or population genomic analyses.

### Adaptability and Physiological Insights

Although the three sequenced *N. moseri* strains are genetically similar, they displayed distinct morphological traits under varying culture conditions (Fig. 1) and slight differences regarding their growth condition tolerance (Fig. 5) and carbon source utilization (Fig. 6; Table 7). However, all strains exhibited strong conidiation on oat medium, making it a reliable choice for conidia collection in future experiments.

The BIOLOG assays revealed that all strains preferentially metabolized sugars, though with noticeable differences in biomass production. TUCIM 5827 consistently showed lower biomass accumulation across all substrates (Fig. 6; Table 7), correlating with its weaker growth on MEX and oat media (Fig. 2). The wide range of metabolized carbon sources suggests a highly versatile and adaptive catabolic system, reinforcing the idea that *N. moseri* is a saprotroph with considerable ecological resilience.

We also observed that γ-aminobutyric acid (GABA) was efficiently metabolized by CBS 164.80 and TUCIM 5827, while TUCIM 5799 did so moderately. This is particularly intriguing as plants produce GABA in response to stress, including fungal infections [58, 59]. While GABA can inhibit the growth of certain plant pathogens [60, 61], the ability of *N. moseri* to utilize GABA may confer an ecological advantage and may be a potential adaptation to an endophytic or epiphytic lifestyle. Further studies are needed to determine whether *N. moseri* can withstand inhibitory GABA concentrations or if it exclusively utilizes it as a nutrient.

The strains also exhibited a certain degree of halotolerance, characterized by optimal growth at low NaCl concentrations and reduced growth at higher concentrations (Fig. 3A). Acidotolerance was observed, with CBS 164.80 and TUCIM 5827 thriving at pH 3, while TUCIM 5799 preferred pH 5 (Fig. 3B). Importantly, the strains demonstrated growth across a broad pH range including even very high pH values, indicating their ability to survive in diverse environmental conditions. Thermotolerance varied among the strains, with TUCIM 5799 being the only one able to grow at 37 °C (Fig. 3C). This observation is significant given the shared habitat of TUCIM 5799 and TUCIM 5827, suggesting a localized adaptation or microevolutionary divergence. In general, *N. moseri* can be classified as a mesophilic species, with strain-specific physiological adaptations that are likely to contribute to its ecological success.

### Mitochondrial genome

The mitochondrial genome of *N. moseri* contains 14 conserved protein-coding genes (Fig. S1), as expected for fungi, with one exception: the *atp8* gene (encoding for the ATP synthase F0 subunit 8) is absent. This gene was presumably transferred to the nuclear genome, as a single gene encoding for a putative ATP synthase subunit can be found in each genome of the three strains (JN550g13373 in *N. moseri* CBS 164.80; JX265g13592 in *N. moseri* TUCIM 5799; JX266g13823 in *N. moseri* TUCIM 5827). Such gene transfers are well-documented in fungi and highlight evolutionary genomic plasticity. This phenomenon may influence mitochondrial function and warrant further investigation into its implications for energy metabolism and strain-specific adaptation.

### Genome mining and metabolic potential

The extensive CAZyme repertoire in *N. moseri* (Table 6) supports the notion of a saprotrophic lifestyle, and indicates metabolic flexibility, allowing *N. moseri* to utilize a variety of complex and simple carbon sources, a trait confirmed by BIOLOG analysis that suggests an oligotrophic lifestyle.

Genome mining for BGCs revealed a striking potential for secondary metabolite production, consistent with its classification in the *Amphisphaeriales* order [8, 9]. *N. moseri* demonstrates a biosynthetic potential comparable to or even exceeding that of fungi known for and studied partly due to their secondary metabolism, such as *Aspergillus* and *Fusarium*. We found that most BGCs are present in all three strains, suggesting a highly conserved secondary metabolite profile, matching the high ANI. In general, secondary metabolites can be useful under certain conditions and contribute to an organism’s fitness. We found BGCs for common metabolites such as choline, the siderophore dimethylcoprogen, and the protective DHN-melanin, which contribute to the basic fitness of *N. moseri*, Additionally, *N. moseri* also possesses BGCs that are most likely responsible for the production of antimicrobial compounds, which can contribute to fitness in competitive situations. Interestingly, we also found BGCs likely to produce brassicicene C and koraiol, and the plant growth hormone gibberellin, suggesting that *N. moseri* might not only be an inert epiphyte, but might directly interact with its plant host. Importantly, for most of the predicted BGCs, we could not detect similar BGCs in the MIBIG 4.0 database, suggesting that further exploration of these BGCs could lead to the discovery of novel compounds with pharmaceutical or agricultural applications. Additionally, future studies using expanded and related taxa will be needed to resolve BGC conservation across the order *Amphisphaeriales*.

## Conclusion

This study provides the first genomic and physiological characterization of *N. moseri*, revealing its exceptional biosynthetic potential and its metabolic versatility. The three sequenced strains exhibit a remarkably high number of biosynthetic gene clusters (BGCs), surpassing those of many fungi within the *Amphisphaeriales*. Genomic analysis also uncovered an extensive repertoire of carbohydrate-active enzymes (CAZymes), supporting its role as a versatile saprotroph or epiphyte capable of degrading a broad array of plant-derived substrates. Ecophysiological assays further demonstrate the species’ adaptability to diverse environmental conditions, including variations in salinity, temperature, and pH, as well as its capacity to metabolize a wide range of carbon sources.

Morphological and genetic analyses challenge the current taxonomy, suggesting that *N. trachycarpi* and *N. urticae* may not be distinct from *N. moseri*. Together, these findings not only expand our understanding of the genus *Neoarthrinium* but also position *N. moseri* as a promising candidate for future biotechnological exploration and drug discovery efforts. The presence of unique, uncharacterized BGCs in the genome provides compelling motivation for further studies into the metabolic products of this species. Overall, this work lays the foundation for integrating *N. moseri* into broader studies of fungal ecology, evolution, and natural product biosynthesis.

## Supplementary Information

Supplementary Material 1.

Supplementary Material 2.

Supplementary Material 3.

Supplementary Material 4.

Supplementary Material 5.

Supplementary Material 6.

Supplementary Material 7.

Supplementary Material 8.

Supplementary Material 9.

Supplementary Material 10.

Supplementary Material 11.

Supplementary Material 12.

Supplementary Material 13.

Supplementary Material 14.

Supplementary Material 15.

Supplementary Material 16.

Supplementary Material 17.

Supplementary Material 18.

Supplementary Material 19.

## Data Availability

The following genomic sequences are available at NCBI GenBank: Rubroshorea johorensis (Dipterocarpaceae, Malvales; DNA BarCode maturase K (matK) with accession no. MF993320.1; The mitochondrial genomes with accession no. MW554918, MW660808, and MW660809; the genome assemblies with accession no. GCA_022829205.1, GCA_022829195.1, and GCA_022829225.1.

## Abbreviations

ANI: Average Nucleotide Identity
BGC: Biosynthetic Gene Cluster
BUSCO: Benchmarking Universal Single-Copy Orthologs
CAZymes: Carbohydrate-Active Enzymes
CE: Carbohydrate Esterase
CYAS: Czapek Yeast Autolysate Agar with Salt
DBCAN: Database for automated Carbohydrate-active enzyme ANnotation
GCF: Gene cluster family
GH: Glycoside Hydrolase
GO: Gene Ontology
GT: Glycosyltransferase
HMMER: Hidden Markov Model Search Tool
ITS: Internal Transcribed Spacer
LSU: Large Subunit Ribosomal RNA
MEA: Malt Extract Agar
MEX: Malt Extract Medium
OUT: Operational Taxonomic Unit
PDA: Potato Dextrose Agar
PKS: Polyketide Synthase
PL: Polysaccharide Lyase
RCDPS: Arginine-Containing Cyclodipeptide Synthase
RiPP: Ribosomally Synthesized and Post-Translationally Modified Peptide
RNA: Ribonucleic Acid
SINE/LINE/LTR, Short/Long: Interspersed Nuclear Elements/Long Terminal Repeat
SEM: Scanning Electron Microscopy
