# Supplementary material for: Genome sequencing and physiological characterization of three Neoarthrinium moseri strains
Source: BMC Microbiol. 2025 Aug 21;25:526. doi: 10.1186/s12866-025-04274-z (PMC12369201; doi:10.1186/s12866-025-04274-z)
Supplement: Supplementary file 1 — Supplementary Material 1. [file 12866_2025_4274_MOESM1_ESM.pdf]

Genome sequencing and physiological characterization of three *Neoarthrinium moseri* strains

Nadine J. Hochenegger[1†], Gabriel A. Vignolle[1,2†], Matthias Schmal[1], Robert L. Mach[1], Astrid R. Mach-Aigner[1], Mohammad Javad Rahimi[1], Chin Mei Chan[3], Feng M. Cai[4], Irina S. Druzhinina[1,5], Christian Zimmermann[1§]

[1] Institute of Chemical, Environmental and Bioscience Engineering, TU Wien, Gumpendorfer Strasse 1a, 1060 Wien, Austria

[2] Center for Health & Bioresources, Competence Unit Molecular Diagnostics, AIT Austrian Institute of Technology GmbH, 1210 Vienna, Austria

[3] Chemical Sciences, Faculty of Science, Universiti Brunei Darussalam, Jalan Tungku Link, Brunei Darussalam

[4] School of Ecology, Sun Yat-sen University, Shenzhen 518107, China

[5] Royal Botanic Gardens, Kew, Kew Green, Richmond, Surrey TW9 3AE, UK

[†] Authors contributed equally to the publication

§Corresponding author:

christian.zimmermann@tuwien.ac.at

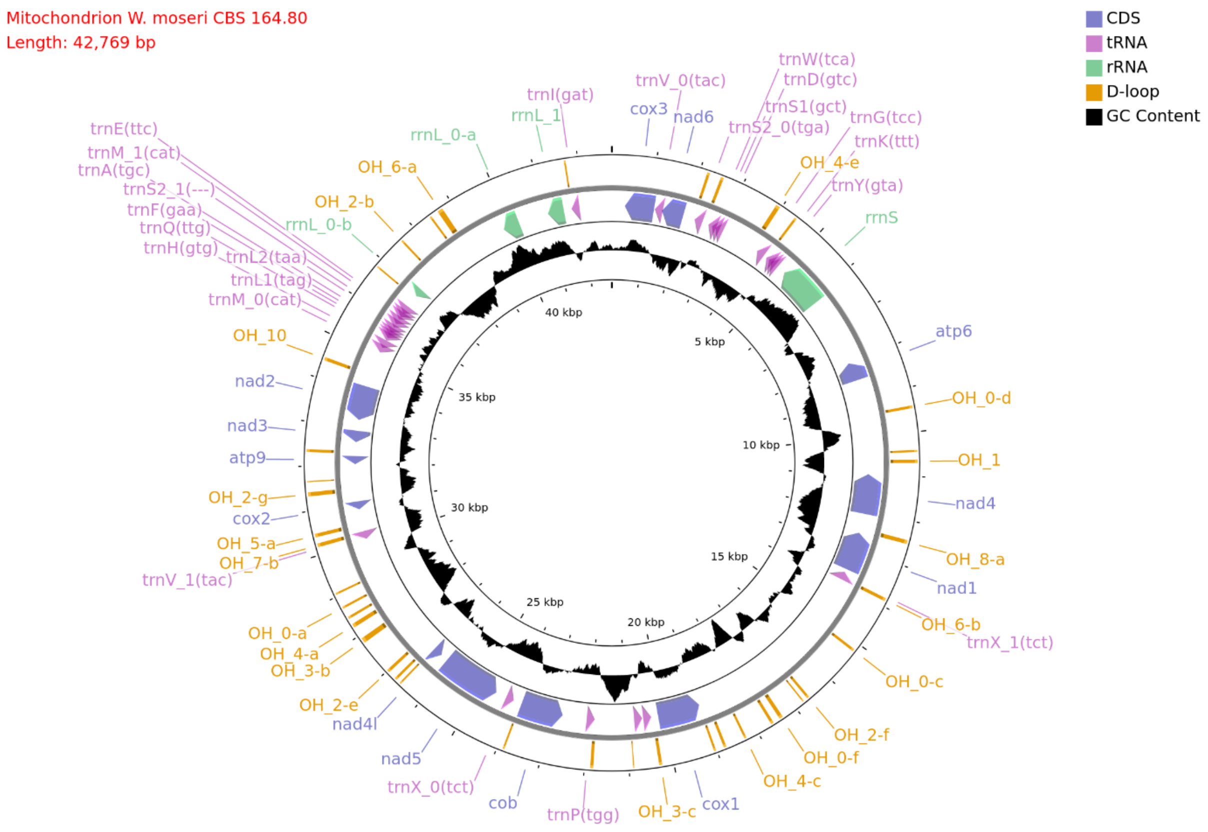

Figure S1. Mitochondrial genome of *N. moseri* CBS 164.80.

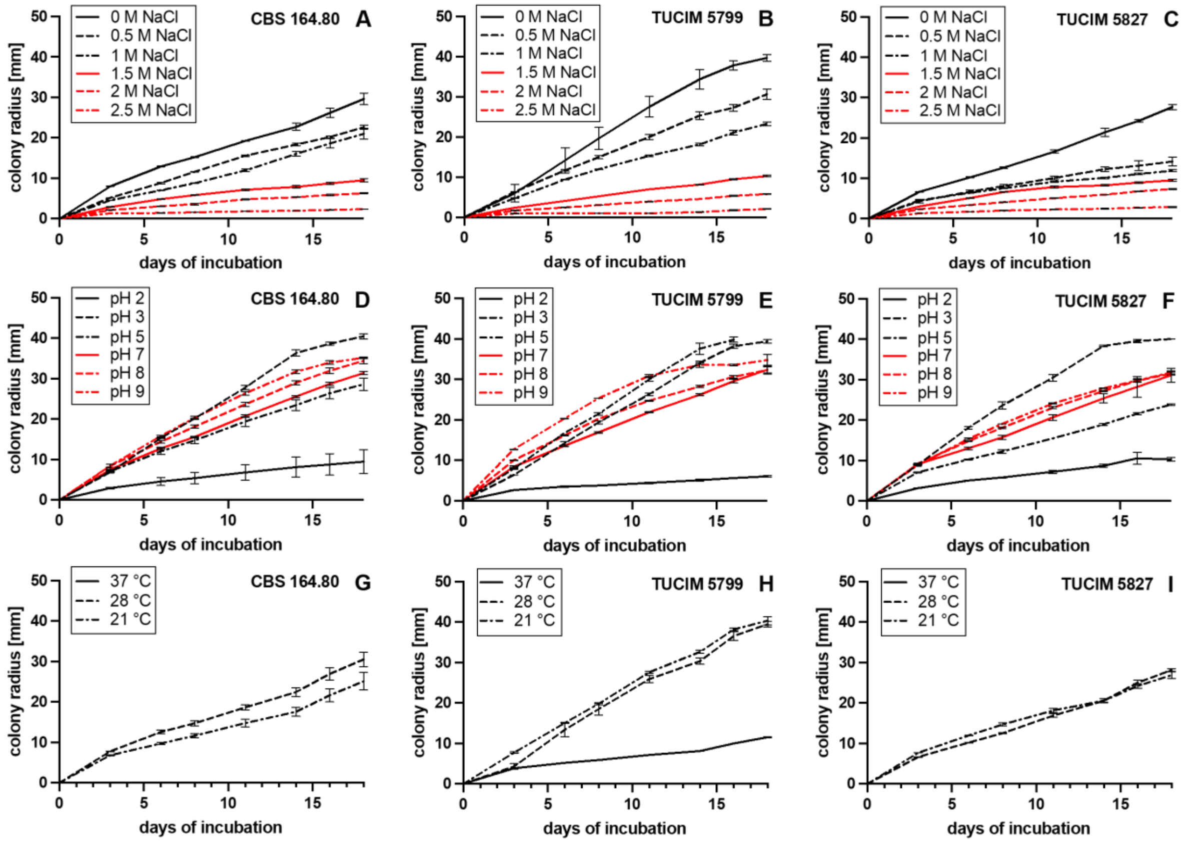

Figure S2. Growth of *N. moseri* CBS 164.80 (A/D/G), TUCIM 5799 (B/E/H), and TUCIM 5827 (C/F/I) on medium containing different NaCl-concentrations (A-C), adjusted to different pH (D-F), and incubated at different temperatures (G-I), respectively. Data show mean of three experiments ± SD

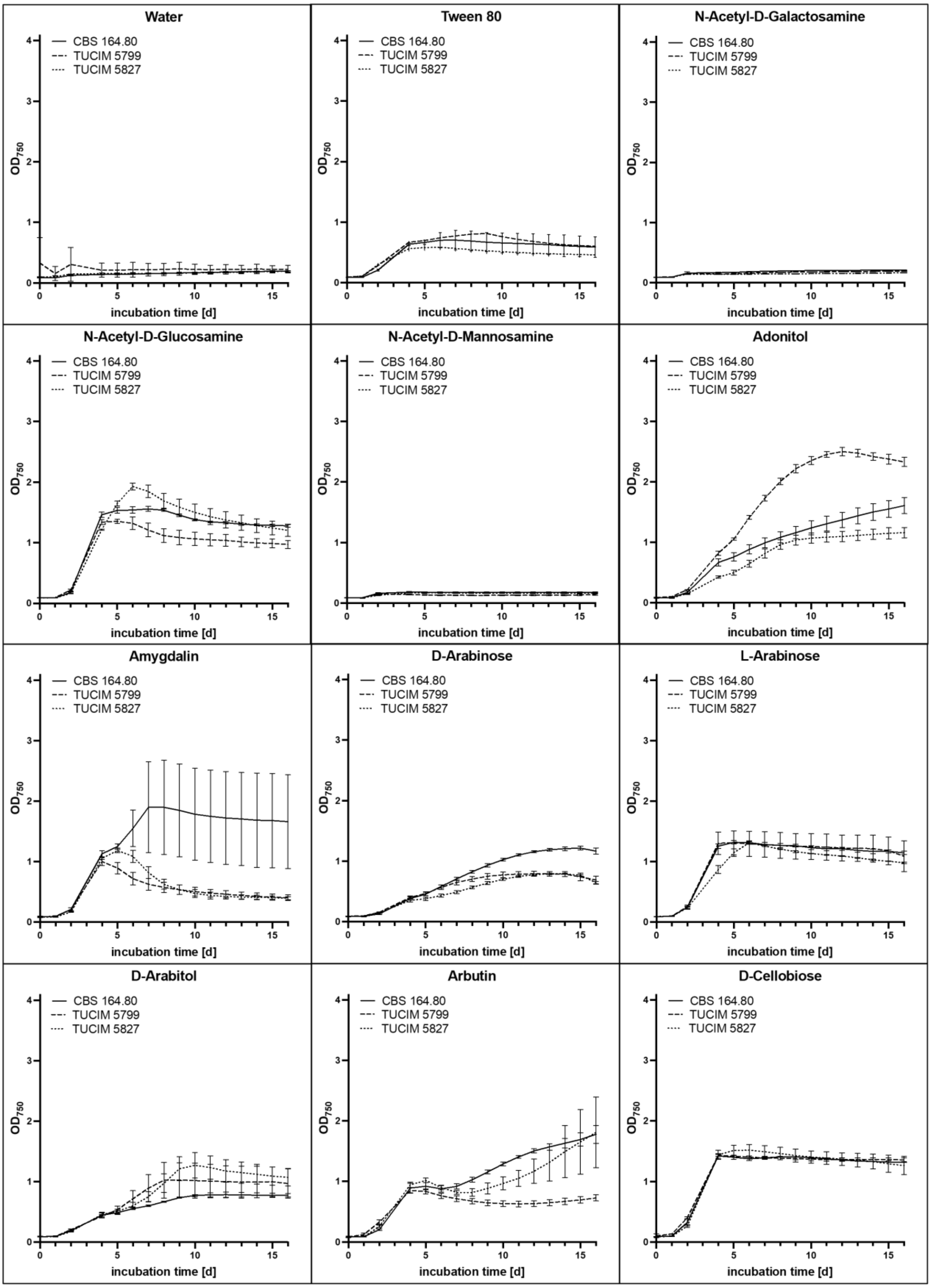
Water
Tween 80
N-Acetyl-D-Galactosamine
N-Acetyl-D-Glucosamine
N-Acetyl-D-Mannosamine
Adonitol
Amygdalin
D-Arabinose
L-Arabinose
D-Arabitol
Arbutin
D-Cellobiose
CBS 164.80
TUCIM 5799
TUCIM 5827
OD_{750}
incubation time [d]
0
5
10
15
1
2
3
4

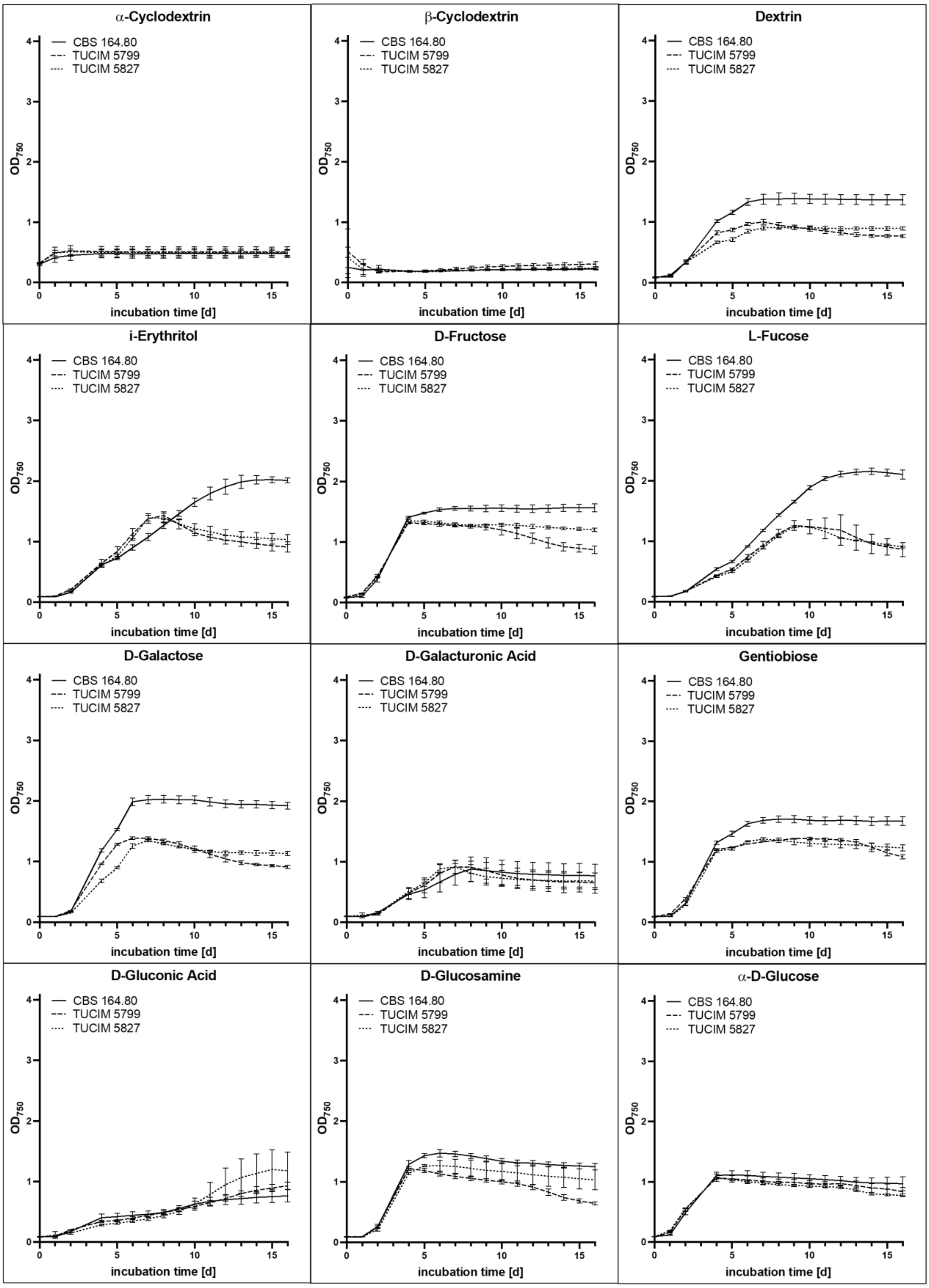

Figure S4. Growth of *N. moseri* CBS 164.80 (A/D/G), TUCIM 5799 (B/E/H), and TUCIM 5827 (C/F/I) on different carbon sources in the BIOLOLG assay.

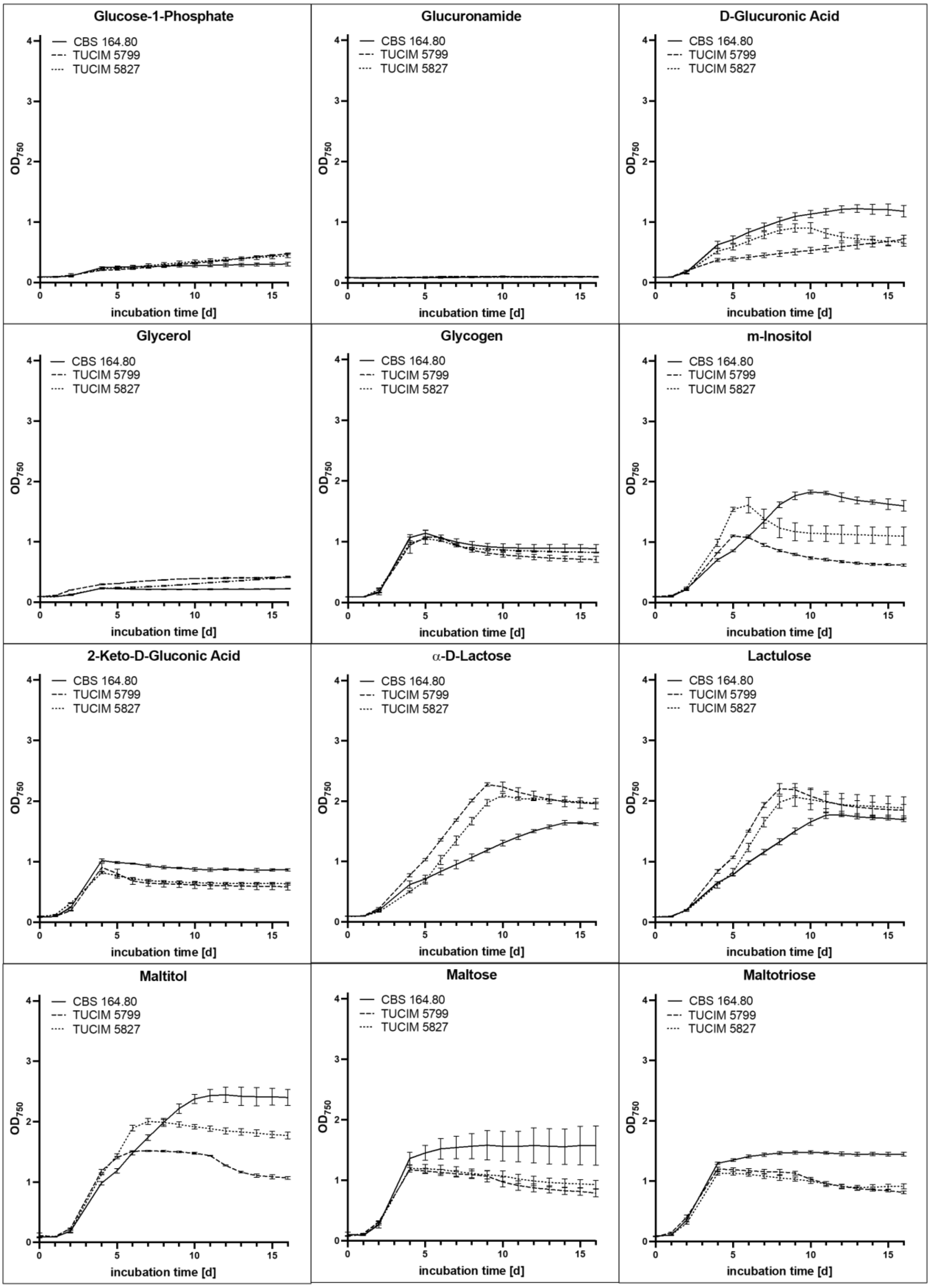

Figure S5. Growth of *N. moseri* CBS 164.80 (A/D/G), TUCIM 5799 (B/E/H), and TUCIM 5827 (C/F/I) on different carbon sources in the BIOLOLG assay.

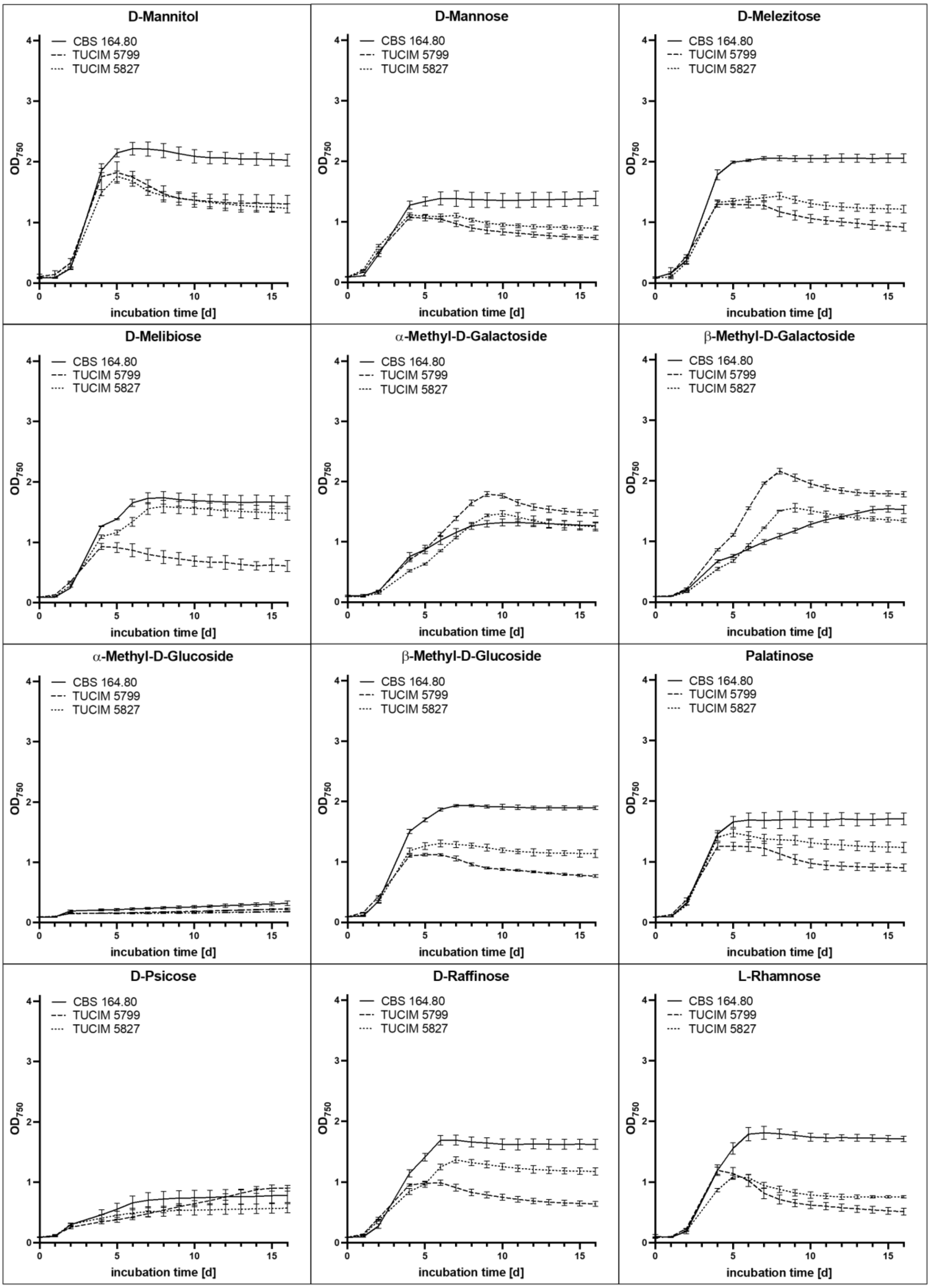

Figure S6. Growth of *N. moseri* CBS 164.80 (A/D/G), TUCIM 5799 (B/E/H), and TUCIM 5827 (C/F/I) on different carbon sources in the BIOLOLG assay.

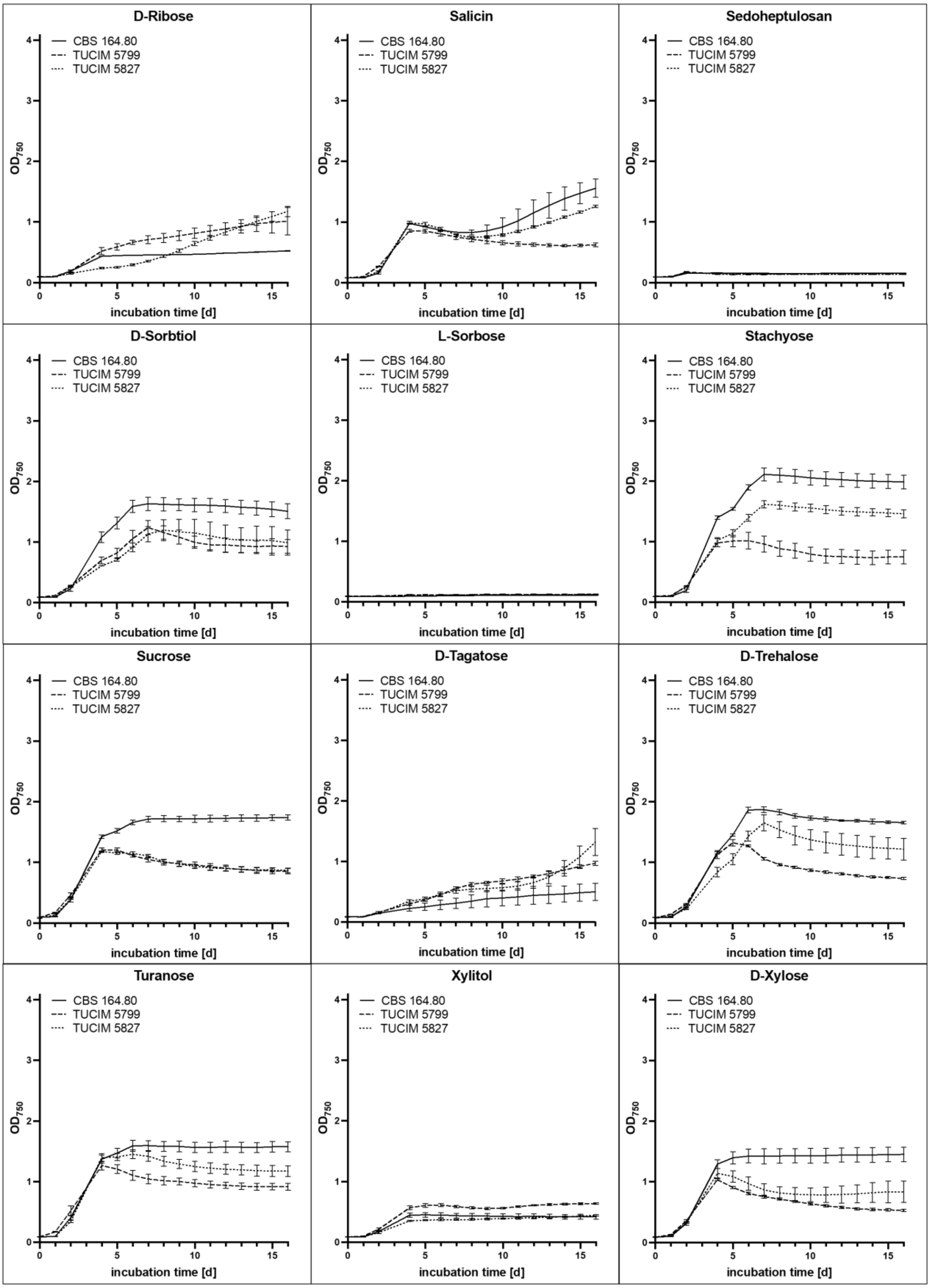

Figure S7. Growth of *N. moseri* CBS 164.80 (A/D/G), TUCIM 5799 (B/E/H), and TUCIM 5827 (C/F/I) on different carbon sources in the BIOLOLG assay.

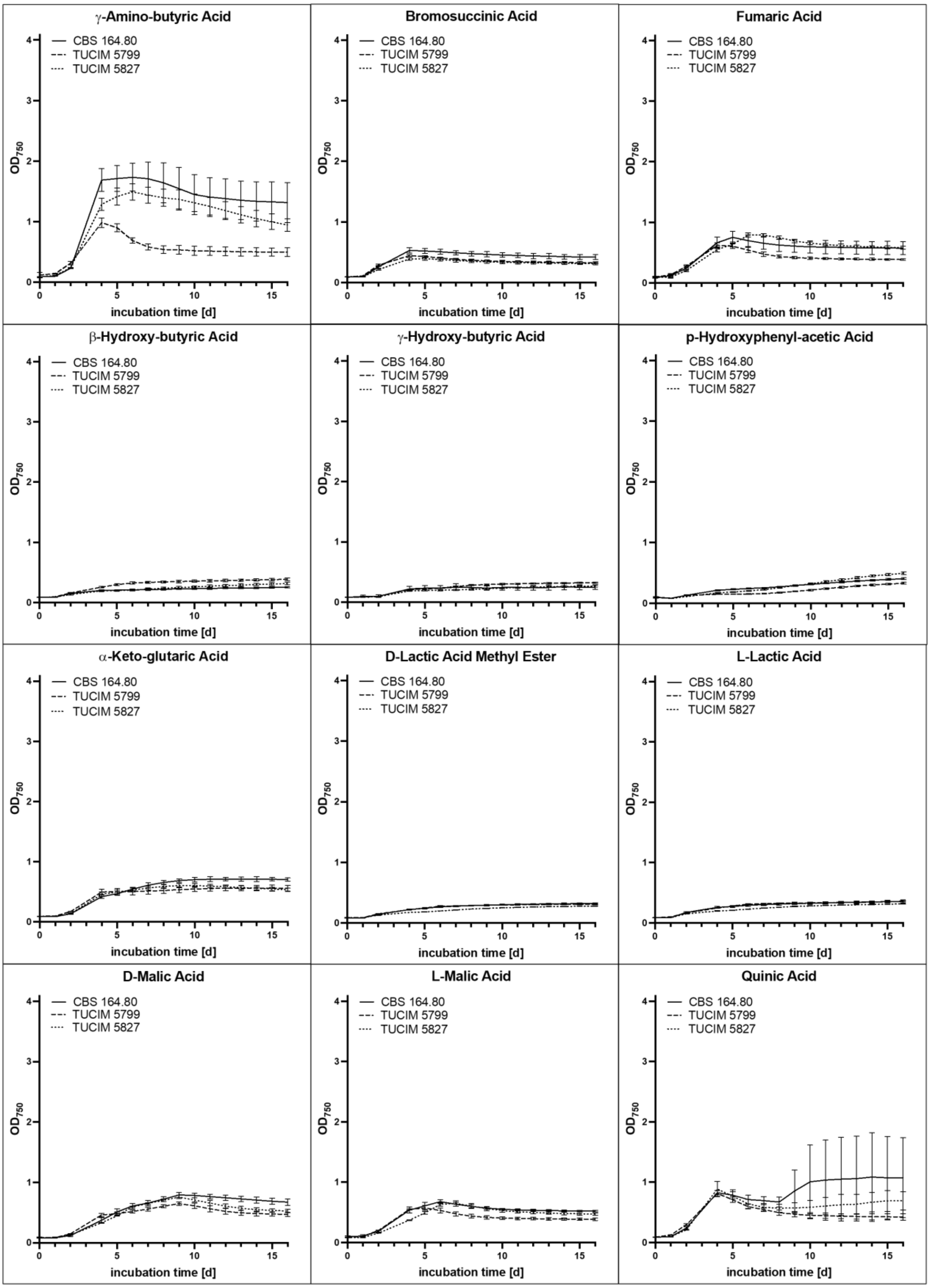

Figure S8. Growth of N. moseri CBS 164.80 (A/D/G), TUCIM 5799 (B/E/H), and TUCIM 5827 (C/F/I) on different carbon sources in the BIOLOLG assay.

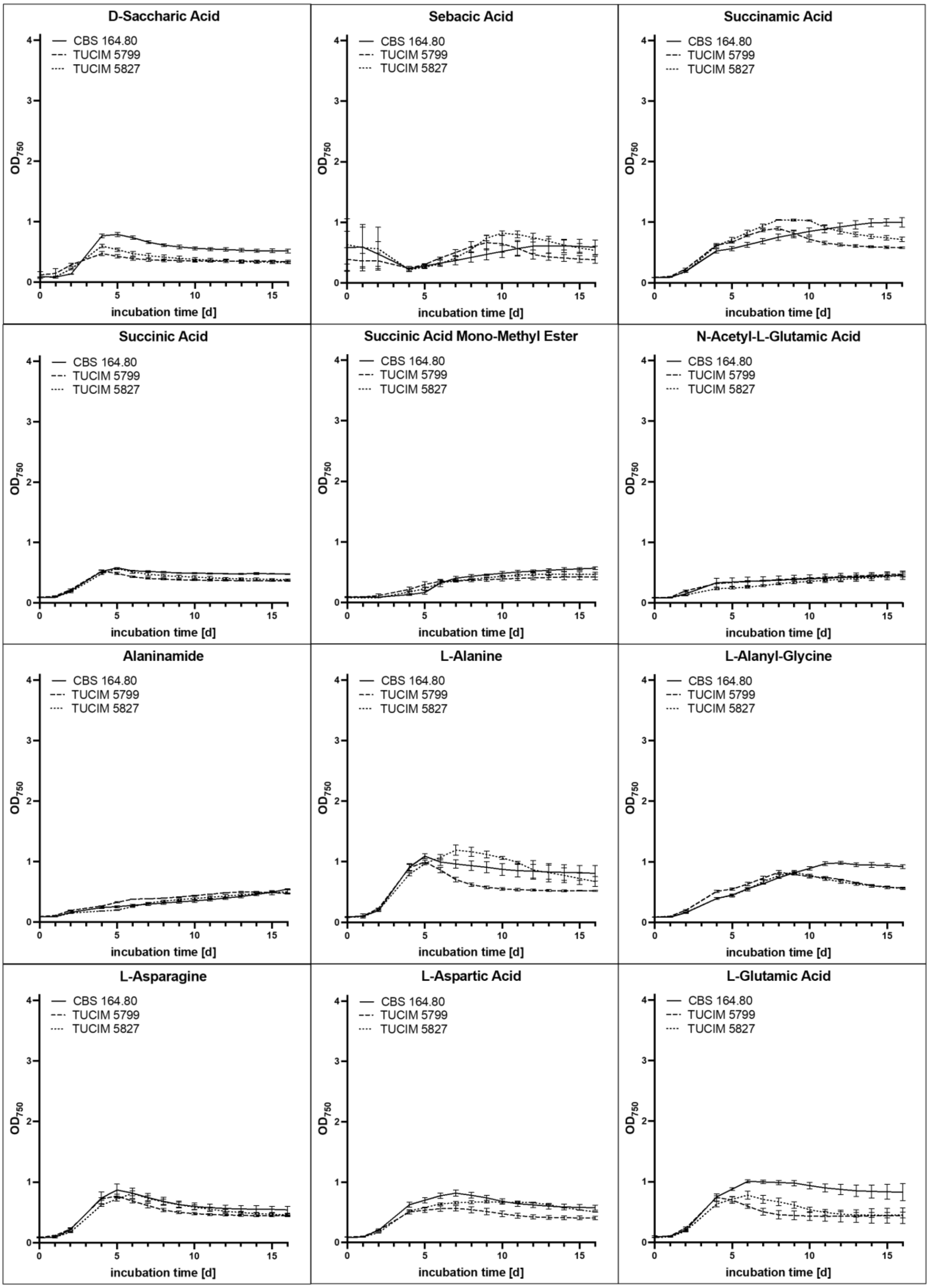

Figure S9. Growth of *N. moseri* CBS 164.80 (A/D/G), TUCIM 5799 (B/E/H), and TUCIM 5827 (C/F/I) on different carbon sources in the BIOLOLG assay.

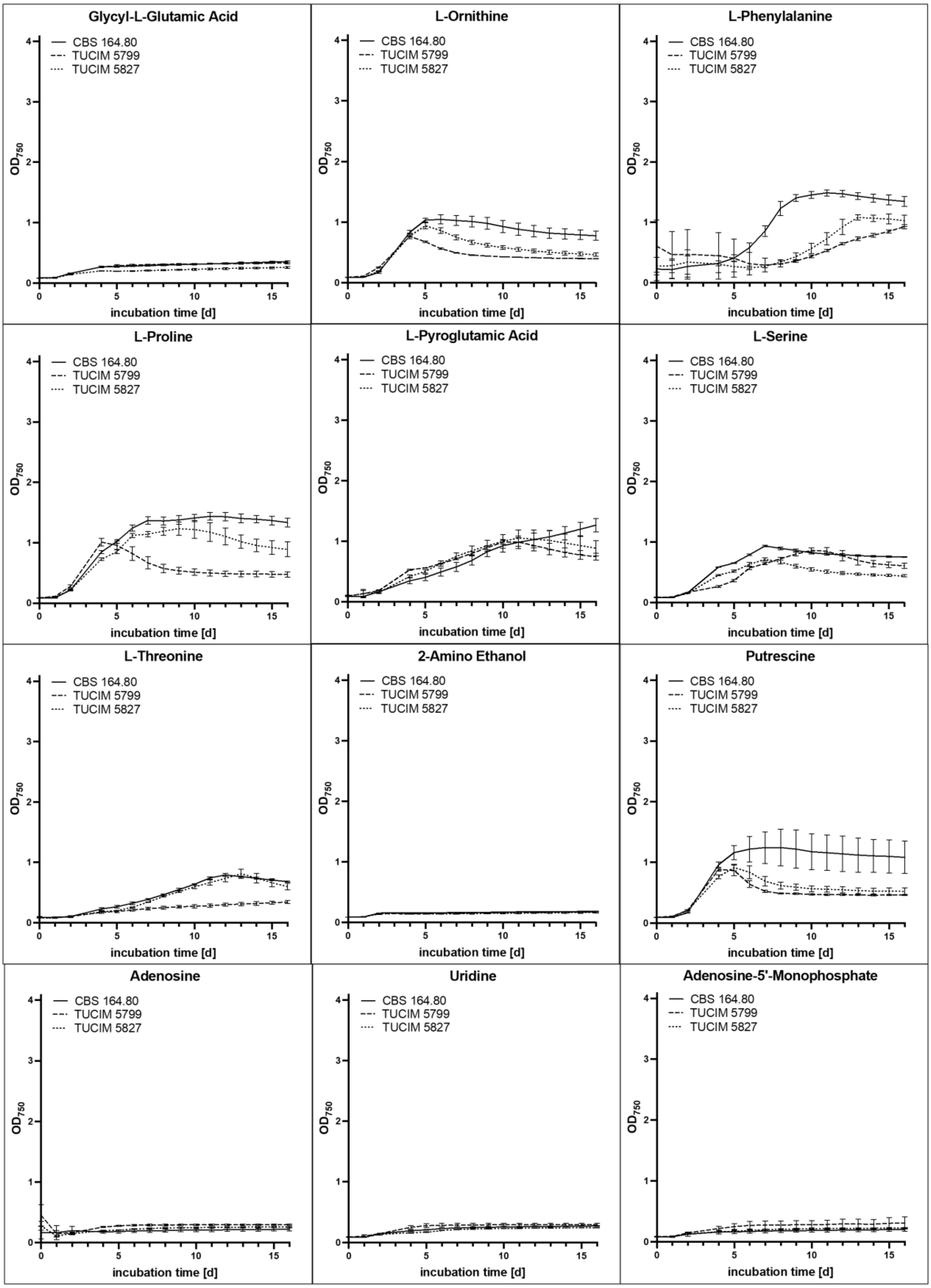

Figure S10. Growth of *N. moseri* CBS 164.80 (A/D/G), TUCIM 5799 (B/E/H), and TUCIM 5827 (C/F/I) on different carbon sources in the BIOLOLG assay.

Table S1. Genome assembly characteristics.

Genome	CBS 164.80	TUCIM 5827	TUCIM 5799
Assembly size (bp)	43,702,215	46,154,457	44,394,130
G+C content (%)	52.77	52.65	52.66
Scaffolds (>= 0 bp)	230	2730	693
Scaffolds (>= 1000 bp)	193	609	221
Largest scaffold (bp)	2,337,669	1,719,970	2,329,648
N50 (bp)	506,940	462,712	764,765
L50 (scaffolds)	26	30	17
N's per 100 kbp	2.33	2.17	1.81
Complete BUSCO (%)	100.00	100.00	100.00
Partial BUSCO (%)	0.00	0.00	0.00

Table S2. Average nucleotide identity (ANI) between the *N. moseri* strains.

Genomes compared	ANI
CBS 164.80 : TUCIM 5799	99.0276
CBS 164.80 : TUCIM 5827	99.0091
TUCIM 5799 : TUCIM 5827	99.1092
