# Supplementary material for: Genome sequencing and physiological characterization of three Neoarthrinium moseri strains
Source: BMC Microbiol. 2025 Aug 21;25:526. doi: 10.1186/s12866-025-04274-z (PMC12369201; doi:10.1186/s12866-025-04274-z)
Supplement: Supplementary file 16 — Supplementary Material 16. [file 12866_2025_4274_MOESM16_ESM.zip › BiGSLICE/output/app/views/layout.html.j2]

BiG-SLiCE v{{ g["version"] }}


{% include "navigation.html.j2" %}

{% include "navigation.html.j2" %}

{% block content %}
{% endblock %}

{% include "modal.html.j2" %}
{% include "copier.html.j2" %}
