# Supplementary material for: Genome sequencing and physiological characterization of three Neoarthrinium moseri strains
Source: BMC Microbiol. 2025 Aug 21;25:526. doi: 10.1186/s12866-025-04274-z (PMC12369201; doi:10.1186/s12866-025-04274-z)
Supplement: Supplementary file 19 — Supplementary Material 19. [file 12866_2025_4274_MOESM19_ESM.zip › BiGSCAPE/output/index.html]

BiG-SCAPE v1.1.8


Biosynthetic Genes Similarity Clustering and Prospecting Engine  
Version 1.1.8

Networks:


Runs:

|  |  |  |
| --- | --- | --- |
|  |  |  |

Found BiG-SCAPE useful? Please consider citing our paper:
